# Recent advances in applications of nanoparticles and decellularized ECM for organoid engineering

**DOI:** 10.1016/j.mtbio.2025.102274

**Published:** 2025-09-04

**Authors:** Sang-Ji Lee, Jae-Yong Cho, Tae-Hyun Heo, Dae Hyeok Yang, Heung Jae Chun, Jeong-Kee Yoon, Gun-Jae Jeong

**Affiliations:** a Institute of Cell and Tissue Engineering, College of Medicine, The Catholic University of Korea, Seoul, 06591, Republic of Korea; b Department of Systems Biotechnology, Chung-Ang University, Anseong-Si, Gyeonggi-Do, 17546, Republic of Korea

**Keywords:** Organoids, Nanoparticles, Decellularized extracellular matrix, Tissue engineering

## Abstract

Organoids have emerged as a transformative *in vitro* platform, offering reliable recapitulation of human tissue architecture and function compared to conventional two-dimensional (2D) cultures. Concurrently, engineered nanoparticles (NPs) have been integrated into organoid systems to enhance scaffold functionality and expand their application in drug delivery, toxicity screening, and disease modeling. Furthermore, decellularized extracellular matrix (dECM) has attracted wide attention for its application in organoid culture, as it provides tissue-specific biochemical and mechanical cues that more closely resemble the native niche, thereby promoting organoid maturation. This review summarizes recent studies that explore how NPs and dECM contribute to the growth and maturation of organoids. It further discusses their applications in therapeutic development and disease modeling, as well as emerging strategies toward refined organoid platforms. Lastly, we outlined how the combined utilization of NPs and dECM may further improve organoid research by enhancing both structural and functional complexity. Together, these approaches support the advancement for developing multifunctional organoid models with broad applicability in disease modeling, therapeutic screening, and regenerative medicine.

## Graphical abstract

**Figure:**
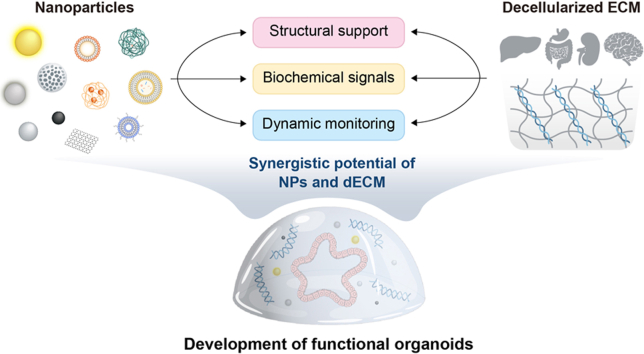

## 1 Introduction

Organoids have revolutionized *in vitro* modeling by enabling the generation of three-dimensional (3D) mini-organs that recapitulate structural and functional properties of native tissues. Conventional two-dimensional (2D) cultures, which have been the standard for decades, lack spatial complexity and cell-to-cell interactions that are crucial for natural organ development and homeostasis. Organoids, in contrast, are derived from tissue-specific or pluripotent stem cells (PSCs) using precisely formulated cocktails of growth factors and supportive scaffolds [1,2]. Under these optimized conditions, the cells self-organize into 3D architectures that exhibit organ-specific features, such as apicobasal polarity, compartmentalization, and multilineage differentiation [3,4]. Due to these advantages, organoids are increasingly utilized not only as biomimetic alternatives to 2D cultures but also as ethically favorable partial substitutes for animal models [5,6]. They have been applied across various biomedical fields and are particularly valuable for studying complex disease processes [7]. For instance, they have proven useful in investigating pathophysiology, therapeutic responses, and drug safety in disease-specific contexts, such as neurodegenerative disorders, liver fibrosis, and cancer [[8], [9], [10], [11], [12]]. Despite these promising features, current organoid systems still face challenges in fully recapitulating the complexity and functionality of native tissues, highlighting the need for further refinement in their structural and environmental design [13].

Concurrently, advances in nanotechnology have introduced a range of engineered nanoparticles (NPs)—nanoscale materials typically ranging from 1 to 100 nm in size—that possess tunable physicochemical properties for biomedical applications [14,15]. These include inorganic (e.g., gold, silver, iron oxide), carbon-based (e.g., carbon nanotubes, graphene oxide), polymeric (e.g., nanocellulose, poly(L-lactic acid)), and lipid-based (e.g., extracellular vesicles, lipid nanoparticles) formulations [16,17]. Given their high surface-area-to-volume ratio, ease of functionalization (e.g., ligand or peptide conjugation), and ability to encapsulate diverse therapeutic or diagnostic agents, NPs have shown significant promise in therapeutic fields such as regenerative medicine and oncology [[18], [19], [20], [21]]. Importantly, when applied to organoid models, these features help overcome key limitations in complex 3D structures, such as restricted nutrient diffusion, inadequate mechanical support, and limited spatial control in imaging or drug delivery [2,22,23]. Furthermore, NP-integrated organoid platforms have shown increasing utility in translational applications, ranging from targeted therapy and biosensing to disease modeling and personalized treatment strategies [[24], [25], [26]].

**Fig. 1:**
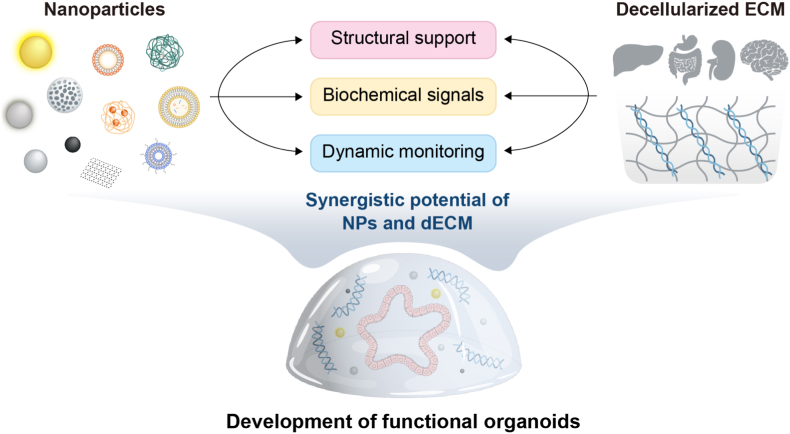
Schematic illustration depicting the synergistic roles of nanoparticles (NPs) and decellularized extracellular matrix (dECM) in development of organoids through structural support, biochemical cues, and dynamic real-time monitoring.

Although nanoparticles have extended the multifunctional capabilities of organoids, there remains a considerable gap in their ability to fully recapitulate the native microenvironment. Organoids require an extracellular matrix (ECM) that provides not only mechanical support but also essential biochemical cues that guide cell differentiation, polarization, and tissue organization [[27], [28], [29]]. Recently, the decellularized extracellular matrix (dECM) has emerged as a promising strategy to resolve the challenges associated with NP-based approaches in organoid culture [[30], [31], [32], [33]]. By removing cellular components and nucleic acids from native tissues, dECM retains essential ECM proteins, glycoproteins, and bioactive factors, providing a tissue-specific microenvironment that better supports *in vitro* tissue development and physiological functionality [34,35]. Unlike synthetic or commercial matrices such as Matrigel, dECM contains a repertoire of organ-specific signals and microstructures that are difficult to replicate artificially. Indeed, several studies have shown that culturing organoids in dECM not only leads to more reliable architectural development but also promotes enhanced functional maturation, as evidenced by increased albumin secretion in hepatic models and improved electrophysiological activity in cerebral models [36,37]. Considering these advantages, dECM can serve as a complementary strategy to nanoparticle-based systems, potentially addressing their biological limitations (Fig. 1).

This review provides a comprehensive overview of recent investigations into the application of nanoparticles and decellularized ECM in organoid research. We highlight how these materials have been employed to enhance the growth, maturation, and functional performance of organoids across various bioengineering strategies. Furthermore, we examine emerging evidence supporting the synergistic integration of NPs and dECM, emphasizing their potential to overcome current limitations and drive the development of more biomimetic and clinically translatable organoid models.

## 2 Nanoparticles (NPs) in organoid research: Recent progress and applications

**Fig. 2:**
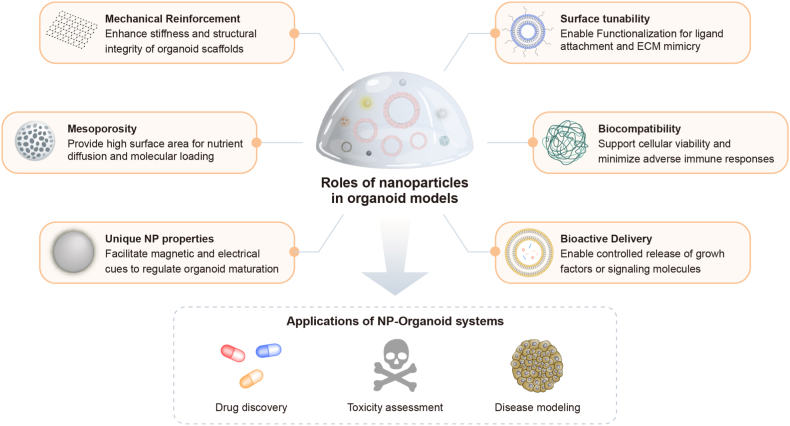
Schematic overview of the NP applications in organoid models.

Nanoparticles (NPs) have developed in parallel with organoid technologies, offering complementary solutions to challenges faced in each field. Nanostructured materials enhance the integrity of organoids through their structural, electrical, and physicochemical properties, while various engineered NPs have been utilized to improve organoid development and physiological functions [23,38]. Conversely, organoid models have introduced organotypic platforms that bridge the gap between preclinical testing and clinical translation in nanomedicine [25,[39], [40], [41]]. While recent work by Shi et al. [22] primarily explored the roles of nanomaterials in regulating stem cell fate and leveraging nanotechnologies to construct 3D microenvironments for personalized medicine, this review shifts focus toward the functional applications of nanoparticles in organoid systems. Specifically, we cover how various types of nanoparticles contribute to the growth and maturation of organoids, as well as their translational potential in therapeutic delivery, toxicity assessment, and mechanistic studies (Fig. 2).

### 2.1 Nanoparticles for enhanced organoid growth and maturation

**Fig. 3:**
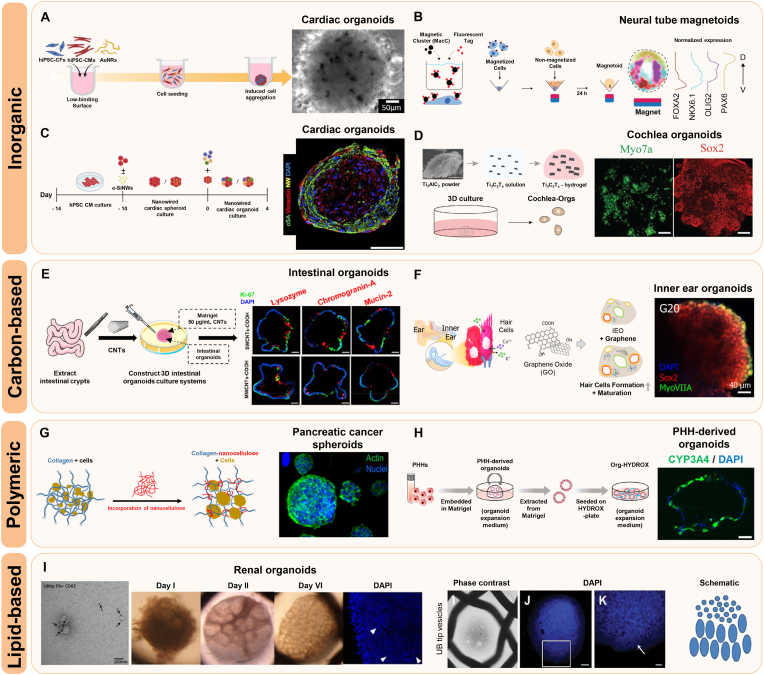
Representative examples of nanoparticles (NPs) used for organoid development. **A** Application of gold nanoribbons (AuNRs) for the formation of hiPSC-derived cardiac organoids. Reprinted with permission from Ref. [52] Copyright 2023, Royal Society of Chemistry. **B** Generation of human neural tube magnetoids and the expression patterning of neuronal markers. Reprinted with permission from Ref. [58] Copyright 2023, Springer Nature. **C** Sequential seeding of hPSC-derived cardiomyocytes and supporting cells onto an electrically conductive silicon nanowire (e-SiNW) scaffold. Reprinted with permission from Ref. [67] Copyright 2023, American Association for the Advancement of Science. **D** Fabrication of Ti_3_C_2_T_x_MXene hydrogel and representative confocal images showing hair cell and supporting cell markers in cochlear organoids. Reprinted with permission from Ref. [71] Copyright 2022, John Wiley and Sons. **E** Construction of intestinal organoids using single-walled (SWCNTs) and multi-walled carbon nanotubes (MWCNTs). Reprinted with permission from Ref. [75] Copyright 2021, American Chemical Society. **F** Generation and characteristics of graphene-inner ear organoids. Reprinted with permission from Ref. [80] Copyright 2023, American Chemical Society. **G** Nanocellulose-based collagen matrix supporting pancreatic cancer cell spheroids. Reprinted with permission from Ref. [97] Copyright 2024, American Chemical Society. **H** Incorporation of primary human hepatocyte (PHH)-derived organoids into a biodegradable 3D nanofiber hydrogel (HYDROX). Reprinted with permission from Ref. [101] Copyright 2024, Springer Nature. **I** Formation and cellular organization of renal organoids via internalization of exosomes derived from a ureteric bud (UBtip) cell line. Reprinted with permission from Ref. [105] Copyright 2018, John Wiley and Sons.

Organoids require microenvironments that closely recapitulate physiologically relevant conditions, including appropriate scaffold composition, mechanical stiffness, and spatiotemporally controlled molecular signals [1,2]. Nanoparticle-based approaches are regarded as promising strategies, providing mechanical reinforcement, controlled delivery of bioactive molecules, and improved cell-cell or cell-ECM interactions [[42], [43], [44]]. Through deliberate design and surface functionalization, nanoparticles can optimize the organoid niche, ultimately supporting more robust growth and functional maturation. To address these multifaceted requirements, different categories of nanoparticles—including inorganic, carbon-based, polymeric, and lipid-based—have been investigated for their utility in organoid culture (Table 1) (Fig. 3).

**Table 1:** Nanoparticles used for the enhanced growth and maturation of organoids.

Type	NPs	Characteristics	Functions in organoid models	Organoid types	Refs
Inorganic	AuNPs	Biocompatible; surface-conjugated with BDNF	Promote neuronal differentiation	Cerebral organoids	[51]
	AuNRs	Improved cellular aggregation; scaffold-free organoid formation	Enhance electrical conductivity and intercellular interactions	Cardiac organoids	[52]
	Gold and iron oxide MNPs	Magnetic properties	Facilitate 3D levitation and innervation	Salivary glands organoids	[56,57]
	Fe3O4 MNPs	Magnetic properties	Guide asymmetric tissue growth and neural tube development	Neural tube magnetoids	[58]
	Fe3O4 MNPs	Magnetic properties; thermoresponsive	Reduce cryo-damage during freeze-thaw cycles	Heart organoids	[62]
	Silicate NPs	Mesoporous; low toxicity	Promote B cell proliferation and GC reactions	B cell follicle organoids	[65]
	Silicon nanowires	Electrically conductive	Improve electrical pacing and cardiac repair	Cardiac organoids	[67]
	Ti_3_C_2_T_x_ MXene	Electrically conductive; mechanical flexible	Promotes hair cell formation via mTOR signaling	Cochlear organoids	[71]
Carbon-based	CNTs	Mechanical reinforcement; high surface area	Accelerate ECM degradation and mechanical signaling	Intestinal organoids	[75]
	SCACs	Peptide-assembled; resembled with SWCNTs	Enhance ESC adhesion and neurogenesis	Forebrain organoids	[76]
	GO	Electrically conductive; high mechanical strength	Boosts hair cell maturation and function	Inner ear organoids	[80]
Polymeric	NC	TEMPO-oxidized; biocompatible	Supports hepatocyte differentiation	Liver organoids	[93]
		TEMPO-oxidized; RGD-functionalized	Promotes epithelial differentiation and maturation	Small intestinal organoids	[94,95]
		Collagen-nanocellulose hybrid; thermoresponsive	Supports epithelial organization	Intestinal organoids	[96]
		Collagen-nanocellulose hybrid	Enhances uniform spheroid growth	Pancreatic cancer cell spheroids	[97]
	HYDROX	PSar-PLLA based nanofiber hydrogel; biodegradable	Facilitates hepatic differentiation and long-term culture	PHH-derived organoids	[101]
Lipid-based	Exosomes	Kidney UB cell line derived vesicles; biocompatible	Enhance nephron patterning and survival	Renal organoids	[105]

Abbreviations: AuNPs, gold nanoparticles; AuNRs, gold nanoribbons; MNPs, magnetic nanoparticles; CNTs, carbon nanotubes; SCACs, single-chain atomic crystals; GO, graphene oxide; NC, nanocellulose; BDNF, brain-derived neurotropic factor; GC, germinal center; mTOR, mammalian target of rapamycin; SWCNT, single-walled CNT; ESC, embyonic stem cell; UB, ureteric bud; PHH, primary human hepatocyte.

#### 2.1.1 Inorganic

Inorganic nanoparticles possess distinctive features, such as tunable structures, facile functionalization, and stable physicochemical properties, which collectively support improved organoid growth and maturation [[45], [46], [47]]. Gold nanoparticles (AuNPs) are widely used for their strong biocompatibility and versatile surface chemistry, designating them as ideal nanocarriers for the delivery of organoids [44,[48], [49], [50]]. AuNPs conjugated with brain-derived neurotrophic factor (BDNF) have been reported to enhance neuronal differentiation in cerebral organoids [51]. By incubating 25-day-cultured cerebral organoids with these BDNF-conjugated AuNPs, researchers achieved deep nanoparticle penetration without cytotoxicity, leading to upregulated expressions of neuronal differentiation genes and more mature cerebral organoids. In addition to zero-dimensional AuNPs, one-dimensional gold nanoparticles, such as nanoribbons (AuNRs), have been introduced for organoid systems. In a cardiac organoid model, AuNRs have been synthesized using a bi-surfactant system consisting of cetrimonium bromide (CTAB) and sodium oleate (NaOL), followed by PEGylating to improve biocompatibility and reduce cytotoxicity (Fig. 3A) [52]. When introduced to cardiac organoids, these AuNRs promoted cellular aggregation and scaffold-free formation of organoids, while enhancing electrical conductivity and intercellular interactions, thereby contributing to the development of functionally mature cardiac organoids.

Magnetic nanoparticles (MNPs) have also emerged as powerful tools for organizing 3D tissue constructs [[53], [54], [55]]. Adine et al. assembled innervated secretory epithelial organoids by magnetically levitating cells using gold and iron oxide NPs crosslinked with a poly-L-lysine biopolymer, demonstrating key aspects of salivary gland epithelial morphology and neural innervation [56,57]. Additionally, Abdel Fattah et al. introduced a novel platform termed "magnetoids," where localized magnetic clusters within neural organoids enabled spatiotemporally controlled mechanical stimulation (Fig. 3B) [58]. This internal actuation significantly guided asymmetric tissue growth, cytoskeletal remodeling, and dorsoventral patterning in neural tube development. Beyond their magnetic properties, iron oxide NPs have also been utilized for their thermoresponsive features in cryopreservation [[59], [60], [61]]. In nanowarming systems, Fe_3_O_4_ nanoparticles have been incorporated into cryoprotectants for organoids to reduce osmotic stress and inhibit ice formation during the freeze-thaw process [62]. Researchers investigated that this method significantly preserves cell viability and restores their physiological functions, such as beating ability and contractile force, in heart organoids following freeze-thaw cycles.

Silicon/silica nanoparticles (Si/SiO_2_NPs) have also been applied to organoid culture due to their mesoporous structure, easy availability, and biologically low toxicity [63,64]. To develop *ex vivo* immune organs, Purwada et al. introduced a B cell follicle organoid model by embedding B cells and engineered stromal cells in a gelatin-silicate NP hydrogel, designed to mimic germinal center (GC) reactions typically seen in secondary lymphoid organs [65,66]. In this environment, B cells rapidly upregulated GC markers and underwent antibody class switching, which are hallmarks of GC responses, indicating the potential of silicate-based materials for understanding complex immune processes. Furthermore, electrically conductive silicon nanowires (e-SiNWs) were incorporated into cardiac organoids to enhance electrical pacing and improve therapeutic efficacy for heart transplantation (Fig. 3C) [67]. When applied to ischemia/reperfusion (I/R)-injured rat hearts, the organoids significantly recovered contractile performance and cardiac functions.

In addition to gold, iron oxide, and silicate, other inorganic nanoparticles also show considerable promise for organoid applications. Recently, MXenes, an emerging 2D inorganic materials, have gained attention in tissue engineering due to their distinctive properties, such as high electrical conductivity and mechanical flexibility [[68], [69], [70]]. In a cochlea organoid model, Zhang et al. demonstrated that incorporating Ti_3_C_2_T_x_ MXenes into a Matrigel scaffold modulated the mechanical properties of the hydrogel and enhanced hair cell development via mammalian target of rapamycin (mTOR) signaling (Fig. 3D) [71]. The MXenes-Matrigel also facilitated robust synapse-like connections between hair cells and sensory neurons, suggesting the potential of MXene-based systems for advancing hearing-loss therapies.

#### 2.1.2 Carbon-based

Carbon-based nanoparticles are characterized by mechanical robustness, high surface area, and adjustable chemical properties, all of which can support the process of 3D tissue formation and functional maturation [[72], [73], [74]]. Among them, carbon nanotubes (CNTs)—either single‐walled (SWCNTs) or multi-walled (MWCNTs)—play a remarkable role in intestinal organoids development by modulating both extracellular matrix (ECM) and intracellular metabolism (Fig. 3E) [75]. Researchers have shown that CNTs reduce ECM stiffness by upregulating matrix metalloproteinase (MMP) expression (e.g., MMP2, MMP7, and MMP9), thereby accelerating ECM degradation and enhancing mechanical signaling through the Piezo-p38 MAPK-YAP signaling pathways. Meanwhile, CNTs enter organoids, localize to mitochondria, and boost both oxidative phosphorylation (OXPHOS) and glycolysis, which in turn increases overall ATP production. These changes collectively promote cell proliferation and lineage differentiation in intestinal organoids, although SWCNTs have milder effects than MWCNTs. Furthermore, peptide-assembled single-chain atomic crystals (SCACs), which resembles with SWCNTs, have been applied to enhance human embryonic stem cell (ESC) adhesion and promote neuronal lineage differentiation [76]. During brain organoid development, SCACs have been shown to increase neural markers and drive more robust neuronal differentiation, presumably through providing both physical support and biochemical cues.

Graphene oxide (GO) has emerged as another carbon‐based 2D nanomaterial with high electrical conductivity, mechanical strength, and surface area [[77], [78], [79]]. By facilitating both cell-ECM and cell-cell interactions, GO boosts the differentiation of specialized cell types in inner ear organoids (IEOs), particularly sensory hair cells (Fig. 3F) [80]. In this model, GO incorporation was shown to upregulate gap junction proteins and ECM‐related signals, thereby accelerating hair cell formation and IEO maturity, as well as enabling more sensitive drug screening. These findings emphasize the potential of GO-based hybrids to enhance IEO models by better replicating their native structure and function.

#### 2.1.3 Polymeric

Polymeric nanoparticles, composed of natural or synthetic macromolecules, are widely explored in tissue engineering due to their modifiable physical characteristics and minimal cytotoxicity [[81], [82], [83], [84], [85]]. Nanocellulose (NC) is a promising polymeric material extracted from cellulose, which is derived from various sources, including wood, algae, bacteria, and biomass [[86], [87], [88]]. This biomaterial comprises a range of nanoscale cellulose-based structures, with physical and chemical properties that vary depending on the origin, extraction method, and subsequent surface modifications. Among chemical treatments, 2,2,6,6-Tetramethylpiperidine-1-oxyl (TEMPO) oxidation is one of the most widely used strategies, introducing carboxylic groups onto the surface of cellulose nanofibrils to enhance colloidal stability, enable ionic cross-linking, and promote mechanically stable hydrogel formation [[89], [90], [91], [92]]. TEMPO-oxidized cellulose nanofibrils have been effectively applied in hepatic and intestinal organoid cultures, where they support hepatocyte differentiation and epithelial maturation, respectively (Fig. 3G) [[93], [94], [95]]. Moreover, functionalization with Arg-Gly-Asp (RGD) peptides and cationic cross-linkers such as Ca^2+^ and Mg^2+^ improves cell adhesion and mechanical stability, enabling sustained culture and passaging of organoids. Additionally, collagen-nanocellulose hybrid (COL-NC) scaffolds offer increased spatial uniformity and mechanical strength, promoting spheroid formation and epithelial organization in pancreatic and intestinal organoid models [96,97].

In addition to natural polymer hydrogels, synthetic polymer systems may offer even greater flexibility in engineering the biochemical and mechanical environment of organoids [[98], [99], [100]]. For example, HYDROX, a biodegradable 3D nanofiber hydrogel composed of poly(sarcosine) and poly(L-lactic acid) (PSar-PLLA), was developed for the culture of primary human hepatocyte (PHH)-derived organoids (Fig. 3H) [101]. Despite limited proliferation, HYDROX scaffolds significantly improved the expression of hepatic functional genes, such as *ALB*, *CYP3A4*, *CK8*, and *HNF4α*, and exhibited strong cytochrome P450 enzyme activity comparable to that in PHHs. Furthermore, hepatic organoids in this system maintained their functions for up to 35 days, suggesting their application for chronic hepatotoxicity assessment.

#### 2.1.4 Lipid-based

In recent years, lipid-based nanoparticles, such as extracellular vesicles (EVs), liposomes, and solid lipid nanoparticles, have gained increasing attention in biomedical applications due to their intrinsic biocompatibility, biodegradability, and low immunogenicity [[102], [103], [104]]. While their application in organoid systems remains relatively limited, these nanocarriers hold considerable promises for supporting organoid development. A representative example involves the application of EVs as secondary inductive signals during kidney organogenesis (Fig. 3I) [105]. In this study, exosomes derived from an immortalized ureteric bud (UBtip) cell line were shown to be internalized by metanephric mesenchyme (MM) cells in a kidney organoid model, enhancing cellular organization and MM cell survival. Although some miRNAs associated with Wnt/β-catenin pathway inhibition were identified, the overall exosomal cargo favored activation of this pathway, indicating a supportive role in nephrogenesis. These findings underscore the potential of exosomes in developmental signaling and highlight the need for further research on lipid-based nanoparticles in organoid engineering.

### 2.2 Applications of NP-organoid systems

**Fig. 4:**
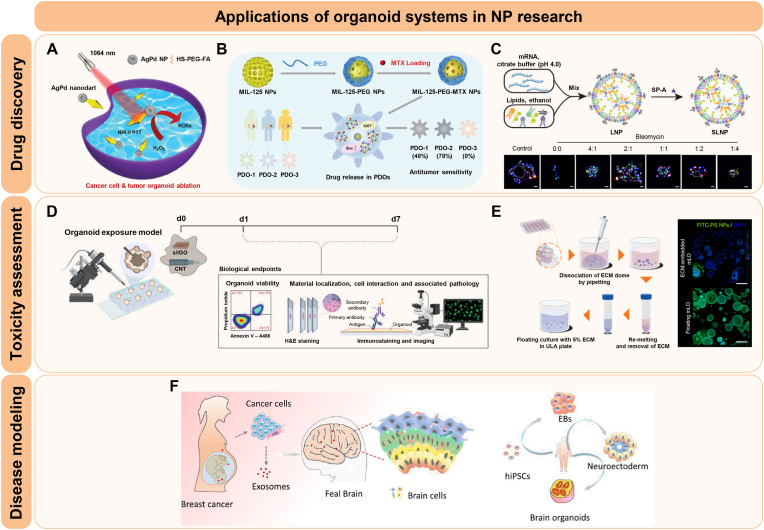
Applications of organoid systems in NP research. **A** Photothermal tumor ablation in cancer organoids using AgPd nanoparticles. Reprinted with permission from Ref. [108] Copyright 2024, John Wiley and Sons. **B** Evaluation of the anti-tumor efficacy of metal-organic framework (MOF) nanoparticles in patient-derived organoids (PDOs). Reprinted with permission from Ref. [107] Copyright 2024, American Chemical Society. **C** Assessment of a lipid nanoparticle (LNP)-based delivery system for pulmonary disease using alveolar organoid models. Reprinted with permission from Ref. [110] Copyright 2024, American Association for the Advancement of Science. **D** Toxicity evaluation of carbon-based NPs via microinjection in a human lung organoid model. Reprinted with permission from Ref. [116] Copyright 2024, Elsevier. **E** A novel method for nanotoxicity testing using floating culture for organoids. Reprinted with permission from Ref. [120] Copyright 2024, American Chemical Society. **F** Modeling breast cancer exosome-mediated fetal neurodevelopmental effects using brain organoids. Reprinted with permission from Ref. [127] Copyright 2022, Springer Nature.

The integration of functional organoids and nanotechnology creates a powerful platform for advancing translational research. Herein, we introduce the biomedical applications of NP-Organoid systems, with a focus on drug delivery and therapeutic strategies, toxicity and safety assessments, and disease diagnosis and mechanistic research within organoid models (Fig. 4).

#### 2.2.1 Drug delivery and therapeutic strategies

Recent advances in nanomedicine have been further accelerated by the emergence of organoid systems, which closely resemble *in vivo* tissue environments for evaluating nanoparticle-based therapeutics [106,107]. These biomimetic models provide suitable tools for investigating drug responses, including therapeutic efficacy, toxicity, and underlying biological mechanisms of nanomedicine—particularly in complex diseases such as cancer and neurodegeneration. These approaches offer deeper insights that are often challenging to obtain using conventional 2D cultures or animal models.

A growing number of studies have demonstrated the potential of NPs in delivering anti-cancer therapeutics using organoid models. For instance, Zhang et al. synthesized multifunctional plasmonic “nanodarts” by depositing AgPd bimetallic tips on gold nanobipyramids and applied them to cancer cells and tumor organoids for photothermal-catalytic combined cancer cell ablation, achieving efficient tumor cell destruction (Fig. 4A) [108]. To further enhance functionality, the researchers selectively coated the AgPd tips with other nanoparticles, including zeolitic imidazolate framework-8 (ZIF-8) and TiO_2_, generating hybrid nanodarts with improved antimicrobial and structural properties. Similarly, nanosized metal-organic framework (MOF) nanoparticles were evaluated for their anti-tumor efficacy in a colorectal cancer (CRC) organoid model (Fig. 4B) [107]. Among three typical MOFs—ZIF-8, ZIF-67, and MIL-125—MIL-125 demonstrated superior biocompatibility in both intestinal and hepatic organoid models. When loaded with methotrexate (MTX), it showed efficient drug loading and release, resulting in significant anticancer effects in patient-derived CRC organoids. In another study, semiconducting polymer nanoparticles functionalized with hyaluronic acid (HA) were developed for CD44-targeted photothermal therapy in CRC organoids [109]. Despite limited tissue penetration, HA coating facilitated targeted retention at the periphery of organoids, allowing efficient heat-induced ablation and reduced tumor viability upon laser activation.

In addition to cancer research, organoid systems have also been applied to explore nanotherapeutics targeting complex diseases that are difficult to model using conventional systems. For idiopathic pulmonary fibrosis (IPF), inhalable mucus-penetrating mRNA-lipid nanoparticles (LNPs) promoted alveolar type 2 (AT2)-to-AT1 epithelial cell differentiation and supported alveolar regeneration, as demonstrated in an alveolar organoid model (Fig. 4C) [110]. This platform also enabled the evaluation of co-delivered *BMP4* and *CYB5R3* mRNAs, which restored mitochondrial function and attenuated fibrotic remodeling.

In myocardial ischemia-reperfusion injury (IRI), Poh et al. reported that mesenchymal stem cell-derived small extracellular vesicles (MSCs-EVs) significantly reduced apoptosis and improved contractile recovery in human cardiac organoids, indicating their cardioprotective effects [111]. Metabolomic analysis further showed that EV treatment modulated unsaturated very long-chain fatty acids (VLCFAs), lipids associated with oxidative stress, thereby reducing cell death and promoting functional recovery of cardiac tissue.

Additionally, in a neurodegenerative disease model, Sol to B-Mica Powder (STB-MP), mica-derived nanoparticles, were applied to Alzheimer's disease (AD) patient-derived cortical brain organoids, resulting in a significant reduction in Aβ plaque accumulation without inducing cytotoxicity [112]. Mechanistically, STB-MP exerted its therapeutic effects by suppressing proinflammatory cytokine levels and γ-secretase activity, while concurrently promoting autophagy through the inhibition of mTOR expression within the AD brain organoid model. Collectively, these findings emphasize the utility of organoid platforms for evaluating nanoparticle-based delivery systems, therapeutic efficacy, and mechanistic action across diverse diseases.

#### 2.2.2 Toxicity and safety assessments

To expand the clinical applicability of nanoparticles, accurate toxicity and safety evaluation are essential. Organoid models are increasingly utilized in nanotoxicity screening due to their ability to replicate native tissue complexity [[113], [114], [115]]. For example, a human lung organoid model was used to evaluate pulmonary response to carbon-based nanoparticles by microinjecting graphene oxide (GO) and multi-walled carbon nanotubes (MWCNTs) into the lumen of the organoids, mimicking inhalation exposure (Fig. 4D) [116]. Consistent with *in vivo* pulmonary fibrosis models, GO exhibited no significant level of toxicity, while MWCNTs induced fibrotic remodeling, evidenced by epithelial thickening and elevated mesenchymal marker expression in the organoid system. These results reveal the relevance of lung organoids in assessing pulmonary toxicity and fibrogenic responses to nanoparticles.

Importantly, organoid models are increasingly used to investigate NP-induced neurotoxicity. For example, Huang et al. applied cerebral organoids from human induced pluripotent stem cells (hiPSCs) to evaluate the neurodevelopmental toxicity of silver nanoparticles (AgNPs) [117]. Exposure to low and high concentrations over a 7-day period revealed that higher doses of AgNPs caused critical cilia morphology disruption, impaired cytoskeletal integrity, and suppressed neuronal marker expression in a dose-dependent manner. While brain organoids represent a promising model for assessing NP-induced neurotoxicity, this study also emphasizes the importance of integrating *in vitro* blood-brain barrier (BBB) models through organ-on-a-chip or 3D-printed microfluidic technologies to address limitations and better simulate *in vivo* neurovascular environments. In another study, hiPSC-derived 3D brain organoids expressing cortical layer proteins were used to assess the neurotoxicity of zinc oxide nanoparticles (ZnO NPs) [118]. In this study, high concentrations of Zn NPs induced cytotoxicity primarily through defective autophagy and intracellular Zn ion accumulation, rather than oxidative stress. Fluorescence micro-optical sectioning tomography demonstrated that the outer organoid layers, directly exposed to NPs, showed reduced LC3B protein expression and increased micronuclei formation, while autophagy inhibition further increased cytotoxicity and Zn ion buildup, indicating the importance of autophagy in mediating ZnO NP-induced neurotoxicity.

Hepatotoxicity evaluation using organoid platforms has also provided valuable insights into NP-induced liver injury. In one study, human liver organoids and a rat convulsion model were utilized to investigate the potential hepatotoxic effects of aspartic acid-coated magnesium oxide nanoparticles (MgO NPs) and the antiepileptic drug valproate, both individually and in combination [119]. The combined exposure induced marked liver toxicity, as evidenced by decreased cell viability, reduced ATP production, elevated ROS levels *in vitro*, and increased serum liver enzyme activities *in vivo*. These findings showed that organoid models can detect the effects of compound-nanoparticle interactions that may not be captured by conventional *in vitro* systems. Building on the use of organoid-based hepatotoxicity assays, Lee et al. developed a novel floating culture system for liver organoids that bypass the limitations of conventional ECM-embedded methods (Fig. 4E) [120]. This method produced uniformly sized organoids with preserved hepatic differentiation markers, while significantly enhancing the internalization of nanoparticles such as gold and polystyrene nanoparticles. Notably, cytotoxic effects of zinc oxide (ZnO) nanoparticles were evident only in floating organoids, as ECM-embedded organoids impeded nanoparticle access and failed to reflect toxicity. These results establish the floating system as a more effective and reliable strategy for standardized safety evaluation of nanoparticles.

In addition to determining cytotoxicity, organoid models can also assess the biocompatibility of NPs by evaluating the maintenance of tissue-specific functions, structural integrity, and inflammatory response. Iqbal et al. synthesized biocompatible magnetic Fe-TiO2 nanorods via solvothermal and thermal decomposition methods, and assessed biocompatibility by both conventional 2D cell culture and 3D organoid models [121]. Similarly, Leite et al. tested the neurotoxicity and biocompatibility of gold nanoparticles (AuNPs) and polylactic acid nanoparticles (PLA-NPs) using human 3D brain models [122]. They demonstrated that AuNPs, particularly PEGylated forms, induced mitochondrial dysfunction, activated oxidative stress genes, and altered cytokine profiles, whereas PLA-NPs maintained cellular physiology in the organoid models. These results show that multicellular 3D brain models are valuable tools not only for evaluating the safety of nanoparticles but also comprehensively assessing their biocompatibility and further indicate that these NPs represent a safer option for CNS drug delivery. Collectively, these studies suggest the versatility of organoid platforms for comprehensive assessment of nanoparticle safety and biocompatibility across diverse organ systems.

#### 2.2.3 Disease diagnosis and mechanistic studies

The application of NP-organoid models is expanding to facilitate the development of novel diagnostic strategies and to elucidate the pathophysiology of complex diseases. For instance, Parkinson's disease (PD) is a progressive neurodegenerative disorder characterized by the accumulation of α-synuclein and subsequent neurotoxicity [[123], [124], [125]]. Consequently, the early and accurate detection of this condition remains a major clinical challenge, emphasizing the necessity for advanced diagnostic strategies that involve sophisticated techniques, such as nanoparticle-based methods and patient-derived organoid systems. Lee et al. developed peptide-imprinted poly(hydroxymethyl 3,4-ethylenedioxythiophene) (PEDOT) nanotube sensors for electrochemical detection of α-synuclein, a key biomarker in PD [126]. The sensing device demonstrated high sensitivity, with a detection limit of 4.0 pM, and were successfully applied to culture medium collected from patient-derived midbrain organoids. This organoid-based system allowed noninvasive, real-time monitoring of α-synuclein expression, supporting its utility in modeling disease-associated protein aggregation and facilitating early diagnostic screening.

In another example, Cui et al. developed a brain organoid-on-a-chip model to study the developmental effects of breast cancer-derived exosomes on the fetal brain (Fig. 4F) [127]. When exposed to exosomes from breast cancer cells (MCF-7), the hiPSC-derived brain organoids exhibited enhanced expression of stemness markers (*OCT4*, *NANOG*) and forebrain development markers (*PAX6*, *FOXG1*), indicating aberrant maintenance of pluripotency and altered regional identity. Subsequent RNA sequencing further revealed the enrichment of genes associated with breast cancer, medulloblastoma, and other tumorigenic pathways. As a result, these findings suggested that the exposure of tumor-derived exosomes may trigger carcinogenic transformation and impair early neurodevelopment. These results imply that exosomal communication from maternal tumors could cause neurodevelopmental risks to the fetus, which highlights the importance of organoid-chip systems in modeling complex tumor-host interactions.

In summary, nanoparticles—spanning inorganic, carbon-based, polymeric, and lipid-based categories—have been extensively integrated into organoid platforms to reinforce structural and biochemical microenvironments. They also enhance electrical and mechanical coupling, and deliver bioactive or therapeutic agents that promote growth, maturation, and functionality related to diseases. These combined systems have demonstrated utility in targeted drug delivery, photothermal and catalytic therapies, standardized nanotoxicity assessment, and precise disease diagnostics. Despite these advances, several challenges constrain the translational potential of NP-organoid systems. First, NP-based scaffolds lack tissue specificity and basement-membrane cues, which limits lineage stabilization and functional maturation [93]. Second, ECM-mimetic niches with tissue-specific stiffness and viscoelasticity are required to recapitulate *in vivo*-like responses and to improve the predictivity of NP safety and efficacy assessment [128]. Third, the transport and retention of nanoparticles are often heterogeneous, with diffusion bottlenecks that drive peripheral accumulation [120]. Incorporating ECM components can mitigate these issues by supplying native proteins and basement-membrane motifs, enabling control over stiffness and viscoelastic properties, and providing similar architecture and poroelasticity that promote more uniform NP penetration and application.

## 3 Decellularized extracellular matrix (dECM): Current results in organoid applications

**Fig. 5:**
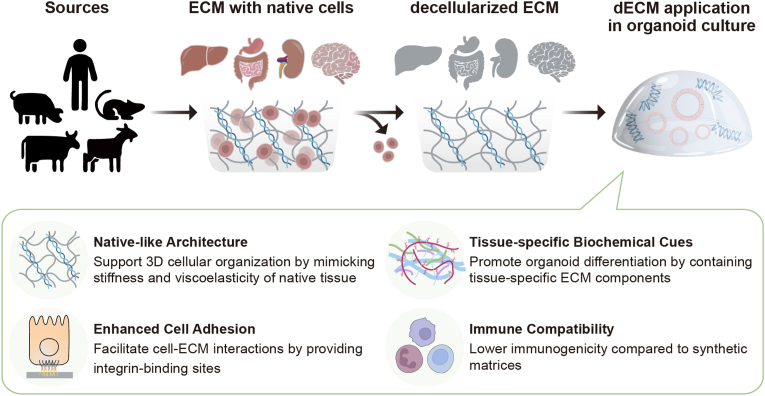
Schematic illustration of dECM-based scaffold engineering for the organoid applications.

As organoid systems advance toward greater functional maturity, decellularized extracellular matrix (dECM) serves as a versatile biomaterial that enhances organoid development and supports broader application in translational research. This section reviews recent results in dECM-based scaffolds for diverse organoid systems, with a focus on tissue-specific applications and bioengineering strategies to improve the maturation of organoids, scalability, and disease modeling (Fig. 5).

### 3.1 Tissue-specific applications of dECM in organoids

**Fig. 6:**
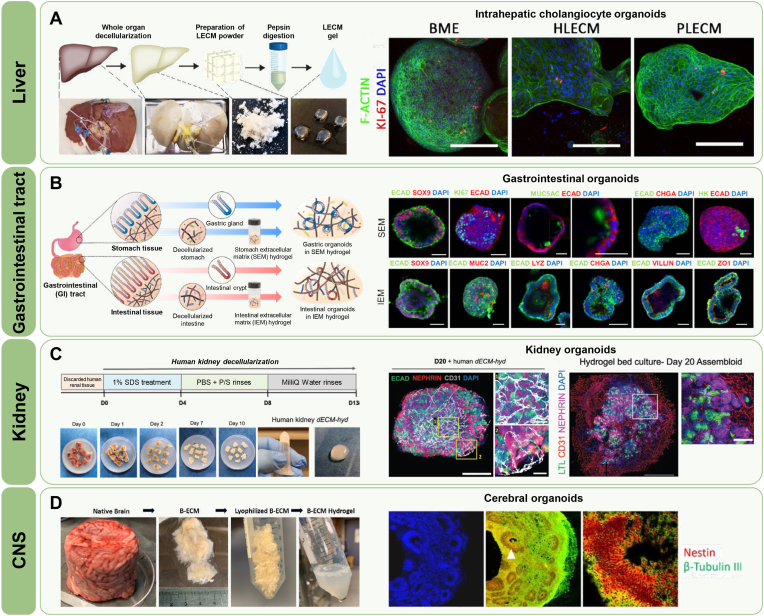
Examples of dECM used for organoid development. **A** Formation of intrahepatic cholangiocyte organoids (ICOs) using liver-derived extracellular matrix (LECM) gels. Reprinted with permission from Ref. [130] Copyright 2022, Elsevier. **B** Development of gastric and intestinal organoids using stomach and intestinal dECM hydrogels. Reprinted with permission from Ref. [132] Copyright 2022, Springer Nature. **C** Generation of kidney assembloids supported by decellularized human kidney ECM hydrogels. Reprinted with permission from Ref. [133] Copyright 2024, John Wiley and Sons. **D** Maturation of cerebral organoids using brain-derived ECM hydrogels. Reprinted with permission from Ref. [136] Copyright 2021, Public Library of Science.

The composition of decellularized ECM varies significantly by tissue and organ of origin, providing a unique biochemical and structural niche for each organoid type. Leveraging tissue-specific dECM enables enhanced lineage commitment, functional maturation, and physiological behavior in organoid cultures. This section summarizes recent studies that employ dECM derived from liver, gastrointestine, kidney, and central nervous system (CNS) to support the development of corresponding organoid systems (Table 2) (Fig. 6).

**Table 2:** Tissue-specific applications of decellularized ECM for organoid development.

Organoid types	Source of dECM	Advantages of dECM	Refs
Human hepatic organoids	Sheep liver dECM	Enhanced hepatic functions (albumin secretion, urea synthesis, CYP3A4 activity) through co-culture with hepatocarcinoma cells, MSCs, and HUVECs in LEMgel	[129]
Human intrahepatic cholangiocyte organoids	Human and porcine liver dECM	Supported organoid formation and maintained hepatic and biliary markers (*ALB*, *KRT19*) comparable to Matrigel despite slower proliferation	[130]
Mouse ductal organoids	Rat liver dECM	Preserved ductal architecture and bile transport functionality; serotonin enrichment	[131]
Human endodermal organoids	SI mucosa dECM	Maintained cell viability and differentiation across multiple passages with preserved ECM proteins and Matrigel-like mechanics	[36]
Human gastrointestinal organoids	Porcine stomach and small intestine dECM	Preserved lineage marker expression and differentiation potential in both gastric and intestinal organoids	[132]
Human kidney organoids	Human and Porcine renal cortex-derived dECM	Promoted nephron-like structure formation and expression of renal and endothelial markers in hPSC-derived kidney assembloids	[133]
Human kidney organoids	Porcine Kidney dECM	Supported vascular network formation and disease modeling (e.g., Fabry nephropathy) via bioactive renal microenvironment	[134]
Human kidney organoids	Mouse kidney dECM	Induced mesenchymal-to-epithelial transition and nephron marker expression (*Nephrin*, *WT1*), supporting CKD drug screening	[135]
Human brain organoids	Porcine brain dECM	Enabled cerebral organoid formation with neuroepithelial structure and expression of *SOX2*, *Nestin*, and *MAP2*	[136]
Human spinal cord organoids	Rat brain dECM	Improved regional marker expression and dorsoventral patterning in spinal cord organoids from hiPSCs	[137]
Human spinal cord organoids	Human placenta dECM	Enhanced dorsoventral organization and laminar marker expression (*PAX6*, *PAX7*, and *FOXA2*), promoting spinal cord-like structure	[138]

Abbreviations: dECM, decellularized extracellular matrix; SI, small intestine; LEMgel, liver-derived dECM hydrogel; CKD, chronic kidney disease.

#### 3.1.1 Liver organoids

Decellularized liver-derived scaffolds offer tissue-specific biochemical and mechanical cues that support the growth and maturation of hepatic organoids. Saheli et al. developed a sheep liver-derived dECM hydrogel (LEMgel), prepared using SDS and Triton X-100, which exhibited suitable pore size and viscoelasticity for hepatic organoids [129]. When co-cultured with hepatocarcinoma cells, mesenchymal stem cells (MSCs), and human umbilical vein endothelial cells (HUVECs), the resulting hepatic organoids showed enhanced hepatic functions, including increased albumin secretion, urea production, and CYP3A4 enzyme activity. Cholangiocytes, another type of liver epithelial cells, have been used to generate intrahepatic cholangiocyte organoids (ICOs) in hydrogels derived from human and porcine liver dECM (HLECM and PLECM) (Fig. 6A) [130]. Despite a slight decrease in proliferation rate in LECM, these collagen-rich hydrogels supported the formation of organoids and maintained hepatic and biliary marker expression (e.g., *ALB*, *KRT19*) comparable to Matrigel. Similarly, rat liver-derived dECM scaffolds were used to generate functional ductal organoids (FDOs) from primary mouse cholangiocytes, preserving biliary architecture and ECM proteins [131]. These scaffolds supported polarized duct-like structures with active bile transport, and metabolomic analysis revealed serotonin enrichment, suggesting a role in biliary development and scaffold bioactivity.

#### 3.1.2 Gastrointestinal organoids

Decellularized gastrointestinal (GI) tissue-derived hydrogels provide organ-specific extracellular matrices that support the formation and differentiation of gastric and intestinal organoids. A porcine small intestine (SI) dECM hydrogel processed by detergent-enzymatic treatment retained key ECM proteins, including collagens, elastin, and glycosaminoglycans (GAGs), and exhibited gelation kinetics and rheological properties comparable to Matrigel [36]. This matrix enabled the culture of human endodermal organoids, such as stomach and small intestinal organoids, and maintained their viability and the expression of differentiation markers over multiple passages. Likewise, stomach- and intestine-specific ECM hydrogels (SEM and IEM), prepared by non-ionic detergent (Triton X-100) decellularization, preserved tissue-specific biochemical composition and exhibited a higher elastic modulus than those prepared using an ionic detergent (sodium deoxycholate; SDC) (Fig. 6B) [132]. Both gastric and intestinal organoids cultured in these hydrogels expressed lineage-specific markers comparable to or higher than those observed in Matrigel, while maintaining consistent stemness and differentiation potential.

#### 3.1.3 Kidney organoids

Kidney dECM hydrogels, by providing a native-like renal microenvironment, contribute significantly to the structural maturation of kidney organoids. For example, porcine and human renal cortex dECM hydrogels were shown to support the differentiation of human pluripotent stem cell (hPSC)-derived renal organoids (Fig. 6C) [133]. These hydrogels facilitated the maturation of nephron progenitor cells (NPCs), leading to the generation of kidney organoids with distinct segmented nephron-like structures. Bulk RNA-seq analysis showed that the resulting organoids had elevated levels of both renal differentiation markers and endothelial markers, prompting further investigation into kidney-endothelial assembloid formation to more closely replicated *in vivo* renal architecture. In addition, porcine kidney dECM exhibits mechanical properties suitable for 3D organoid culture [134]. This approach not only enhanced vascular network formation but also enabled disease modeling, such as Fabry nephropathy, suggesting the potential of ECM-based scaffolds. 10.13039/100014337Furthermore, mouse renal extracellular matrix (rECM) gels have been demonstrated to support the differentiation of hiPSC-derived renal organoids [135]. In this model, the mesenchymal-to-epithelial transition and the expression of nephron markers (e.g., Nephrin, WT1) were observed, indicating its potential utility for drug screening in chronic kidney disease (CKD).

#### 3.1.4 CNS organoids

To better understand the functional and pathological complexity of central nervous system (CNS), a range of neural organoid models have been developed. To further promote their maturation and acquisition of human-specific features, dECMs obtained from tissue-specific niches have been employed as supportive scaffolds. Simsa et al. demonstrated that hydrogels prepared from decellularized adult porcine brain extracellular matrix (B-ECM), composed primarily of retained collagens but lacking several brain-specific proteoglycans, could successfully support the formation of human embryonic stem cell (hESC)-derived cerebral organoids (Fig. 6D) [136]. These organoids exhibited comparable expressions of neural progenitor and neuronal markers (e.g., *SOX2*, *Nestin*, *MAP2*) and developed characteristic neuroepithelial structures, indicating that B-ECM may serve as a tissue-specific alternative to Matrigel for the generation of cerebral organoids. In another approach, rat decellularized brain extracellular matrix hydrogel (DBECMH) has been shown to facilitate the generation of hiPSC-derived spinal cord organoids, with enhanced expression of regional spinal cord markers, including both dorsal and ventral patterning genes [137]. Moreover, a novel thermosensitive hydrogel fabricated from decellularized human placenta ECM (DPECMH), enriched with laminin, fibronectin, collagen I, and elastin, provided a human-specific and biocompatible microenvironment for the generation of spinal cord organoids [138]. Compared to Matrigel, organoids cultured in DPECMH exhibited significantly improved dorsoventral organization and increased expression of key laminar markers such as *PAX6*, *PAX7*, and *FOXA2*, supporting the formation of more mature spinal cord-like structures.

Taken together, these findings reveal the critical role of tissue-specific dECM hydrogels in promoting the development of organoid systems that more closely resemble *in vivo* tissue architecture and function.

### 3.2 Advances in dECM-based organoid models

**Fig. 7:**
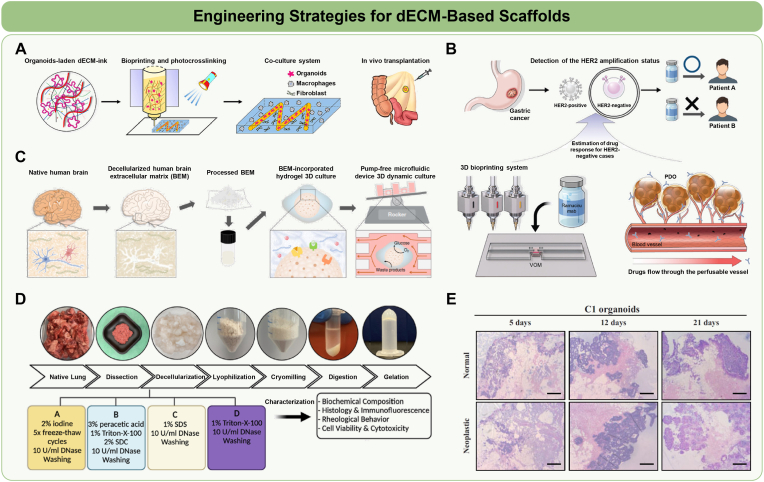
Engineering Strategies for dECM-Based Scaffolds. **A** Fabrication of organoid-laden dECM bioink using a blue light-induced bioprinting method. Reprinted with permission from Ref. [148] Copyright 2022, John Wiley and Sons. **B** Bioprinting and application of vascularized organoid models for screening patient-specific chemotherapeutic responses. Reprinted with permission from Ref. [150] Copyright 2023, John Wiley and Sons. **C** Microfluidic culture of brain organoids using brain dECM hydrogel in a dynamic flow-based device. Reprinted with permission from Ref. [37] Copyright 2021, Springer Nature. **D** Optimization of decellularization protocols for human lung organoids using bovine lung dECM. Reprinted with permission from Ref. [157] Copyright 2023, American Chemical Society. **E** Decellularized matrices derived from normal or neoplastic peritoneum, repopulated with peritoneal metastases (PM)-derived organoids. Reprinted with permission from Ref. [164] Copyright 2022, Oxford University Press.

While dECM-based scaffolds hold great promise for organoid development, several hurdles still limit their widespread adoption. First, native-like mechanical and biochemical cues are often insufficiently replicated, prompting the investigation of advanced engineering approaches—such as matrix remodeling, 3D bioprinting, and microfluidic systems—to better mimic *in vivo* microenvironments and guide tissue organization. Second, the absence of standardized decellularization protocols across tissue sources results in batch-to-batch variability and poor reproducibility of the formation of organoids [[139], [140], [141]]. Finally, tissue- and tumor-specific dECM scaffolds are increasingly paired with patient-derived cells to create organoid disease models and drug-testing platforms that replicate *in vivo* pathology, yet their broader use still depends on precisely tuning ECM composition and mechanical cues while establishing scalable, reproducible fabrication protocols. This section delineates recent bioengineering strategies aimed at overcoming current limitations and enhancing the functional applicability of dECM-based organoid models (Fig. 7).

#### 3.2.1 Advanced engineering strategies and mechanical modulation

To improve the biological relevance and functional performance of organoid systems, scaffold materials must replicate the structural, biochemical, and mechanical properties of native tissues. Recent studies have focused on bioengineering techniques, such as matrix remodeling, 3D bioprinting, and microfluidic integration, combined with tissue-specific decellularized ECM to better guide the differentiation and spatial organization of organoids [[142], [143], [144], [145], [146]]. As one matrix remodeling strategy, Xu et al. introduced thrombospondin-1 (THBS1) into hepatic dECM hydrogels to create a dynamically tunable microenvironment for hepatocyte organoids [147]. This remodeling activated the YAP/TAZ signaling pathway, upregulated reprogramming transcription factors such as KLF4 and SOX2, and induced a progenitor-like state in hepatocytes. Transplantation of these reprogrammed hepatocyte organoids into a hepatectomy mouse model significantly enhanced liver regeneration, demonstrating the therapeutic potential of viscoelastic control in dECM scaffolds.

The integration of tissue-specific dECM into 3D bioprinting platforms has enabled the construction of organoid scaffolds with defined architecture and biomimetic properties. For example, a photocrosslinkable bioink composed of porcine small intestine dECM, gelatin methacrylate (GelMA), and hyaluronic acid supported 3D bioprinting of intestinal organoids while preserving stemness (Fig. 7A) [148]. Layer-by-layer printing allowed co-culture with intestinal subepithelial myofibroblasts and macrophages, which promoted intestinal stem cell (ISC) proliferation and niche-specific differentiation. Upon transplantation into NSG mice, the printed constructs facilitated epithelial regeneration, highlighting the therapeutic potential of dECM-based bioinks. In parallel, Choi et al. and Kim et al. modeled tumor-specific microenvironments by developing vascularized lung and gastric cancer organoids using 3D bioprinting with organ-specific dECM bioinks [149,150]. Choi et al. utilized porcine lung dECM with patient-derived tumor cells, fibrotic lung fibroblasts, and endothelial cells to generate lung cancer organoids that retained oncogenic profiles and resistance to EGFR-targeted therapies under fibrotic conditions [149]. Likewise, Kim et al. employed stomach-derived dECM to bioprint patient-derived gastric cancer organoids (PDOs) with perfusable vasculature, accurately recapitulating the PDO molecular subtype-specific responses to VEGFR2-targeted therapy (Fig. 7B) [150].

Microfluidic systems have been increasingly applied to organoid models to enhance nutrient exchange, reduce hypoxia, and provide physiologically dynamic environments [[151], [152], [153], [154]]. In a brain organoid model, Cho et al. incorporated decellularized human brain ECM (BEM) into a pump-free microfluidic device, providing brain-specific biochemical cues while enhancing organoid viability and reducing batch-to-batch variability (Fig. 7C) [37]. This 3D culture system not only supported structural and electrophysiological maturation of brain organoids but also promoted cell proliferation and reduced apoptosis by enabling continuous nutrient and oxygen exchange through microfluidic perfusion. In liver applications, a microfluidic system was also combined with liver dECM scaffolds to create vascularized liver organoids [155]. Co-cultured with induced hepatic (iHep) cells and endothelial cells under continuous flow, the device enhanced cell-cell and cell-matrix interactions while reducing tissue apoptosis. This combination improved vascularization, hepatic metabolic function, and drug response predictability, supporting its application in pharmacological testing.

#### 3.2.2 Standardization and biomanufacturing of dECM scaffolds

Standardizing decellularization protocols is crucial for ensuring reproducibility and scalability in dECM-based organoid systems. In practice, decellularization is carried out as a sequence of physical, chemical, and enzymatical steps [31,33,156]. Physical methods, such as freeze-thaw cycling, mechanical oscillation, superhigh pressure, and electroporation, promote cellular disruption and reagent exchange while, in thick tissues, preserving three-dimensional and vascular architecture. Chemical reagents are chosen to balance efficacy with ECM preservation: ionic detergents (e.g., SDS, sodium deoxycholate) enable aggressive cell lysis, whereas non-ionic detergents (e.g., Triton X-100, Tween-20) and zwitterionic surfactants (e.g., CHAPS) allow gentler extraction; adjuncts such as hyper/hypotonic buffers, selective acids/bases, alcohols for delipidation, chelators, and oxidants used as needed. Enzymatic finishing with proteases (e.g., trypsin, dispase) and nucleases (DNase, RNase) removes residual adhesions and nucleic acids. Parameters including reagent concentration, temperature, exposure time, and perfusion rate are optimized by each tissue to maximize cellular clearance while preserving collagen/elastin networks, glycosaminoglycans (GAGs), basement-membrane proteins, and matrix-bound growth factors.

However, variability among protocols and source tissues still limits batch-to-batch consistency and scale-up, both of which are critical for the practical use of dECM scaffolds. Kim et al. addressed this challenge by developing gastrointestinal (GI) tissue-derived ECM hydrogels that not only preserved essential ECM components but also demonstrated minimal lot-to-lot variation, providing a reliable alternative to Matrigel for long-term culture or organoids [132]. Their optimized non-ionic detergent-based protocol notably supported the robust formation of gastric and intestinal organoids, indicating the potential of tissue-specific dECM in clinical and industrial settings. In parallel, Kuşoğlu et al. compared four decellularization methods for bovine lung tissue, revealing method-dependent changes in ECM composition, stiffness, and viscoelasticity (Fig. 7D) [157]. By investigating the distinct effects of each protocol on lung cancer cells and patient-derived organoids, this study suggested the need for reproducible methods that preserve key biochemical and mechanical cues. Taken together, both papers reveal the importance of advanced decellularization strategies to achieve consistent, high-quality dECM scaffolds across different types of tissue.

#### 3.2.3 Disease modeling and drug testing

Tissue-specific dECM scaffolds have been increasingly adopted to construct organoid-based disease models that more faithfully recapitulate *in vivo* pathology. In tumor organoid systems, fine-tuning dECM composition is critical for accurately replicating the metastatic microenvironment, which is essential for modeling tumor progression and therapeutic responses [143,[158], [159], [160], [161]]. Extending this concept to organ-specific metastasis, patient-derived cholangiocarcinoma organoids cultured on decellularized human lung or lymph nodes ECM exhibited tissue-specific regulation of epithelial-to-mesenchymal transition (EMT) and cancer stem cell populations. These organoids also showed donor- and tumor-dependent differences in migratory and proliferative behavior, indicating that organ-specific dECM plays a critical role in metastatic outgrowth [162]. Chen et al. developed a liver-derived dECM (L-dECM) scaffold to construct a HepG2-based tumor organoid-like tissue model [163]. The L-dECM scaffold not only enhanced hepatic function but also promoted the EMT, an initial response in tumorigenesis triggered by signals from the surrounding microenvironment. Moreover, the model exhibited differential drug sensitivities, suggesting its utility for *in vitro* anticancer drug screening and EMT-focused studies. Similarly, Varinelli et al. demonstrated that dECM derived from patient neoplastic peritoneum, characterized by increased stiffness and altered composition, enhanced the growth of metastatic colorectal organoids and mimicked *in vivo* tumor behavior (Fig. 7E) [164]. Under simulated hyperthermic intraperitoneal chemotherapy (HIPEC) treatment—a heated chemotherapy approach commonly used for abdominal cancers—this ECM reduced the sensitivity of organoids to standard anticancer drugs, indicating its role in promoting treatment resistance. In a breast cancer model, an autologous dECM derived from MCF-7 cells (CD-dECM), combined with rat tendon collagen type I, provided an oncogenic microenvironment that significantly enhanced the viability and proliferation of organoids [165]. This hydrogel also promoted the formation of larger spheroids and supported the development of breast cancer organoids with improved morphological integrity and epithelial organization.

Beyond oncology, tissue-specific dECM has also been applied to enhance organoid maturation and vascularization. Kidney dECM hydrogels supported the generation of hPSC-derived kidney organoids with extensive vascular networks and their own endothelial cells, as well as more mature glomerular structures with greater similarity to human kidney tissues [134]. This approach also enabled efficient recapitulation of Fabry nephropathy with vasculopathy using GLA-knockout hPSCs, and, upon transplantation into mouse kidney, promoted endothelial cell recruitment from the host and maintained vascular integrity with more organized slit diaphragm-like structures. While substantial progress has been made in tumor-focused dECM-organoid systems, studied addressing other disease types remain limited, highlighting the potential of diverse tissue-specific dECM platforms for developing organoid-based disease modeling.

Collectively, tissue-specific dECM hydrogels and bioinks provide organotypic biochemical and mechanical cues that support lineage specification, polarization, electrophysiological maturation, and durable function across liver, gastrointestinal, kidney, and CNS organoids. When coupled with matrix remodeling, 3D bioprinting, and microfluidic perfusion, dECM scaffolds enable defined architecture, multicellular co-culture, and disease models with greater translational relevance for drug testing and regenerative medicine. However, several challenges still hinder their application in organoid culture. Batch-to-batch variability and growth-factor sequestration can shift biochemical composition and distort signaling gradients, yielding inconsistent exposure [[166], [167], [168]]. Mechanical properties can also drift during culture, complicating controlled transport and functional readouts. Furthermore, limited shelf stability and GMP readiness impede scale-up and standardization. In this context, nanoparticles offer complementary functions, including matrix-anchored or stimuli-responsive delivery to standardize exposure and nanoscale reinforcement to stabilize stiffness and poroelasticity. Accordingly, integrating dECM with NP-based components is a rational next step to improve reproducibility, safety, and clinical translatability, providing the basis for the integration strategies outlined in Section 4.

## 4 Synergistic integration of NPs and dECM: Current challenges and prospects

**Fig. 8:**
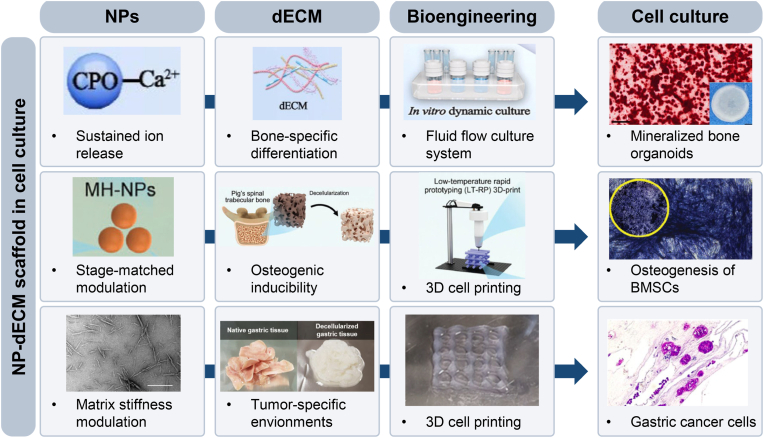
Synergistic Potential of NP-dECM scaffolds in cell culture. Representative examples of NP-dECM hybrid scaffolds applied in cell culture systems, combined with bioengineering technologies to enhance lineage-specific differentiation and functional maturation (Top). A bone-mimetic scaffold integrating nanostructured calcium phosphate oligomers (CPO) and bone-derived dECM facilitates sustained ion release and osteoinductive signaling, supporting *in vitro* mineralization of bone organoids (Middle). Reprinted with permission from (150) Copyright 2025, Elsevier. **C** A 3D-printed hybrid scaffold composed of magnesium hydroxide nanoparticles (MH-NPs), decellularized bone matrix (DBM) microparticles, and PLGA promotes stage-specific modulation of osteogenesis of BMSCs. Reprinted with permission from (152) Copyright 2024, John Wiley and Sons. A tumor-specific NP–dECM scaffold combining cellulose nanoparticles and gastric tissue-derived dECM tunes matrix stiffness to replicate native tumor microenvironments, enabling gastric cancer cell culture in a 3D-bioprinted platform (Bottom). Reprinted with permission from (153) Copyright 2021, Frontiers Media S.A.

Nanoparticles (NPs) and decellularized extracellular matrix (dECM) each advance organoid engineering—NPs by enabling structural support and controlled delivery and sensing, and dECM by supplying tissue-specific biochemical and mechanical cues. Their collaboration can compensate for their respective limitations identified in Sections 2, 3, creating more resembled microenvironments with defined delivery kinetics, stabilized stiffness and poroelasticity, and reliable quantitative measurements. Although the evidence upon this is still emerging, convergent results from various tissue and scaffold engineering point to a clear synergy in mechanical integrity, biocompatibility, and functional versatility, positioning NP-dECM integration as a practical route for next-generation organoid platforms (Fig. 8).

A few studies have explored the combination of tissue-specific dECM and nanoparticles to engineer organoid scaffolds with enhanced mechanical integrity, immunocompatibility, and tissue-specific bioactivity. Gai et al. developed a bone-mimetic hydrogel by assembling bone-derived dECM, salmon DNA, and nanostructured calcium phosphate oligomers (CPO) into a composite matrix tailored for the formation of bone organoids [169]. Designed to emulate the native bone niche, this hydrogel created a synergistic microenvironment that supported osteogenic and angiogenic differentiation of bone marrow-derived mesenchymal stromal cells (BMSCs), driven by the osteoinductive properties of dECM, structural reinforcement from 10.13039/100026054DNA, and sustained calcium ion release from 10.13039/100007298CPO. Consequently, this scaffold enabled *in vitro* mineralization and *in vivo* vascularization, highlighting the translational potential of NP-dECM synergy in organoid engineering.

Beyond current organoid models, several NP-dECM scaffolds developed in tissue engineering research exhibit characteristics that could be translated to organoid culture and maturation. For instance, silver nanoparticle (AgNP)-funtionalized xenogeneic pancreas dECM scaffolds demonstrated reduced immunogenicity and promoted vascular integration *in vivo* [170]. As a cross-linking agent, AgNPs contributed antibacterial, anti-inflammatory, and collagen-binding properties, While the pancreas-derived ECM provided tissue-specific biochemical cues, together supporting mechanical stability and inducing M2 macrophage polarization. In addition, Yuan et al. engineered a 3D-printed hybrid scaffold incorporating magnesium hydroxide nanoparticles (MH-NPs), decellularized bone matrix microparticles (DBM-MPs), and polylactic acid-glycolic acid (PLGA) to recreate a osteoimmune niche [171]. This construct enabled stage-specific modulation of inflammation, angiogenesis, and osteogenesis, offering insights for future strategies in bone organoid research. In another approach, Kim et al. generated a hybrid bioink using cellulose nanoparticles (CN) and gastric dECM to tune matrix stiffness and replicate a tumor-specific environment for gastric cancer cell culture [172]. While not yet applied to organoids, such NP-dECM systems reveal the versatility and future potential of these scaffolds across a wide range of organoid applications.

In addition to providing structural support, NPs offer functional advantages that expand the analytical capabilities of dECM-based organoid systems. In a representative study, Cho et al. combined brain-derived decellularized extracellular matrix (BEM) with a microfluidic device to improve the maturation of human brain organoids [37]. To monitor oxygen dynamics within the organoids, they introduced Pt(II) meso-tetra(pentafluorophenyl)porphine (PtTFPP)-conjugated poly(urethane acrylate nonionomer) (PUAN) nanoparticles as phosphorescent oxygen sensors. This enabled real-time spatial mapping of oxygen distribution and demonstrated improved oxygen penetration under microfluidic conditions. These findings provide evidence for how NP-ECM integration can be harnessed not only for structural enhancement but also for real-time physiological assessment, broadening the utility of organoid platforms in functional studies.

Collectively, while current evidence on NP-dECM synergy in organoid systems remains scarce, converging findings from related scaffold-engineering studies suggest several plausible molecular bases, including NP-mediated modulation of biochemical signaling, sustained release of bioactive ions or molecules, and nanoscale architectural tuning that enhances ECM-cell receptor engagement. Exploring these mechanisms in organoids will be a promising direction for future research and will guide the rational design of hybrid scaffolds with maximized synergistic effects. These scaffolds could enhance structural reinforcement, dynamic sensing, immune modulation, and tissue-specific guidance, thereby improving organoid maturation for applications in drug testing and regenerative medicine.

## 5 Conclusion and future perspectives

Organoid technology has revolutionized *in vitro* modeling by enabling the previse replication of human tissue architecture and function. Nanoparticles (NPs) and decellularized extracellular matrix (dECM) have independently advanced in this field: NPs offer structural integrity, precise control over biochemical delivery, and real-time sensing, while dECM provides tissue-specific biochemical and mechanical cues that conventional matrices lack. Recent efforts combining NPs with dECM have begun to demonstrate synergistic benefits, resulting in multifunctional scaffolds that support organoid maturation, facilitate physiological monitoring, and expand analytical versatility. These NP-dECM systems are currently being explored across various bioengineering applications and show considerable promise for future translation into advanced scaffolds for organoid culture.

Translating NP-dECM hybrid platforms into clinical and industrial use requires overcoming several key challenges. Scalability remains a major hurdle, as the preparation of dECM is labor-intensive, variable in biochemical composition and mechanical properties across laboratories [31,33,156], and the incorporation of NPs demands precise control over size distribution, surface chemistry, and loading efficiency to ensure reproducibility [22,38]. Achieving GMP standards will require automated, high-throughput manufacturing pipelines capable of producing large quantities with consistent performance. Clear understanding of the regulatory pathways for these hybrid biomaterial-drug systems is also essential to guide preclinical and clinical studies. Importantly, in personalized medicine, combining patient-specific ECM with tailored nanoparticles can better replicate individual disease characteristics, improve prediction of therapeutic responses, and thereby enhance the efficiency of clinical transition. Addressing these issues through harmonized protocols, validated analytical methods, and early regulatory engagement will be critical to developing NP-dECM organoid scaffolds from experimental tools to clinically relevant platforms.

In addition to these translational considerations, the continued evolution of NP-dECM organoid platforms will depend on the application of cutting-edge technologies that can resolve current limitations and expand their functional scope. Recent developments in nanoparticle engineering have enabled the design of materials with controlled size, shape, and surface properties, facilitating precise delivery biosensing, and structural reinforcement in organoid systems [22,25,38,173]. Notably, emerging strategies are being explored to overcome the obstacles of toxic nanoparticles for broader biomedical use, including biodegradable coatings, bioinspired surface modifications, and controlled-release construction that minimize off-target effects [174,175]. Furthermore, applying computational modeling and artificial intelligence (AI) with organoid research, such as AI-driven scaffold design, *in silico* prediction of NP-cell interactions, and automated analysis of functional assays, provides powerful opportunities to accelerate optimization and expand the range of NP-dECM applications [[176], [177], [178]].

All together, these innovations will not only address current barriers but also unlock new capabilities for NPs and dECM in organoid models, enabling more efficient drug discovery, disease modeling, and precision medicine. With ongoing technological progress, NPs-dECM integration holds high potential for advancing organoid systems into clinically and industrially applicable platforms. As these hybrid scaffolds continue to be refined, organoid models are expected to become core technologies for bridging the gap between experimental research and therapeutic application, ultimately accelerating the translation of innovative biomedical strategies to clinical use.

## CRediT authorship contribution statement

**Sang-Ji Lee:** Writing – review & editing, Writing – original draft, Visualization, Project administration, Investigation, Conceptualization. **Jae-Yong Cho:** Writing – review & editing. **Tae-Hyun Heo:** Writing – review & editing. **Dae Hyeok Yang:** Writing – review & editing. **Heung Jae Chun:** Writing – review & editing. **Jeong-Kee Yoon:** Writing – review & editing. **Gun-Jae Jeong:** Writing – review & editing, Writing – original draft, Visualization, Supervision, Investigation, Funding acquisition, Conceptualization.

## Declaration of competing interest

The authors declare that they have no known competing financial interests or personal relationships that could have appeared to influence the work reported in this paper.

## Data Availability

No data was used for the research described in the article.

## References

[1] Tang X.-Y., Wu S., Wang D. (2022). Human organoids in basic research and clinical applications. Signal Transduct. Targeted Ther..

[2] Yi S.A., Zhang Y., Rathnam C. (2021). Bioengineering approaches for the advanced organoid research. Adv. Mater..

[3] Brassard J.A., Lutolf M.P. (2019). Engineering stem cell self-organization to build better organoids. Cell Stem Cell.

[4] Co J.Y., Margalef-Català M., Monack D.M. (2021). Controlling the polarity of human gastrointestinal organoids to investigate epithelial biology and infectious diseases. Nat. Protoc..

[5] Park G., Rim Y.A., Sohn Y. (2024). Replacing animal testing with stem cell-organoids : advantages and limitations. Stem Cell Reviews and Reports.

[6] Jensen C., Teng Y. (2020). Is it time to start transitioning from 2D to 3D cell culture?. Front. Mol. Biosci..

[7] Yang S., Hu H., Kung H. (2023). Organoids: the current status and biomedical applications. MedComm.

[8] Lancaster M.A., Renner M., Martin C.-A. (2013). Cerebral organoids model human brain development and microcephaly. Nature.

[9] Grenier K., Kao J., Diamandis P. (2020). Three-dimensional modeling of human neurodegeneration: brain organoids coming of age. Mol. Psychiatr..

[10] Wray S. (2021). Modelling neurodegenerative disease using brain organoids. Semin. Cell Dev. Biol..

[11] Tuveson D., Clevers H. (2019). Cancer modeling meets human organoid technology. Science..

[12] Guan Y., Enejder A., Wang M. (2021). A human multi-lineage hepatic organoid model for liver fibrosis. Nat. Commun..

[13] Andrews M.G., Kriegstein A.R. (2022). Challenges of organoid research. Annu. Rev. Neurosci..

[14] Kinnear C., Moore T.L., Rodriguez-Lorenzo L. (2017). Form follows function: nanoparticle shape and its implications for nanomedicine. Chem. Rev..

[15] Verma J., Warsame C., Seenivasagam R.K. (2023). Nanoparticle-mediated cancer cell therapy: basic science to clinical applications. Cancer Metastasis Rev..

[16] Jeong G.J., Castels H., Kang I. (2022). Nanomaterial for skeletal muscle regeneration. Tissue Eng. Regener. Med..

[17] Altammar K.A. (2023). A review on nanoparticles: characteristics, synthesis, applications, and challenges. Front. Microbiol..

[18] Anselmo A.C., Mitragotri S. (2019). Nanoparticles in the clinic: an update. Bioeng Transl Med.

[19] Afzal O., Altamimi A.S.A., Nadeem M.S. (2022). Nanoparticles in drug delivery: from history to therapeutic applications. Nanomaterials.

[20] Gavas S., Quazi S., Karpiński T.M. (2021). Nanoparticles for cancer therapy: current progress and challenges. Nanoscale Res. Lett..

[21] Francis W.R., Liu Z., Owens S.E. (2021). Role of hypoxia inducible factor 1α in cobalt nanoparticle induced cytotoxicity of human THP-1 macrophages. Biomaterials Translational.

[22] Shi Y., Han X., Zou S. (2024). Nanomaterials in organoids: from interactions to personalized medicine. ACS Nano.

[23] Abdel Fattah A.R., Ranga A. (2020). Nanoparticles as versatile tools for mechanotransduction in tissues and organoids. Front. Bioeng. Biotechnol..

[24] Zhao D.-K., Liang J., Huang X.-Y. (2023). Organoids technology for advancing the clinical translation of cancer nanomedicine. WIREs Nanomedicine and Nanobiotechnology.

[25] Mahapatra C., Lee R., Paul M.K. (2022). Emerging role and promise of nanomaterials in organoid research. Drug Discov. Today.

[26] Liang J., Zhao D.-K., Yin H.-M. (2025). Combinatorial screening of nanomedicines in patient-derived cancer organoids facilitates efficient cancer therapy. Nano Today.

[27] Tortorella I., Argentati C., Emiliani C. (2022). The role of physical cues in the development of stem cell-derived organoids. Eur. Biophys. J..

[28] Hussey G.S., Dziki J.L., Badylak S.F. (2018). Extracellular matrix-based materials for regenerative medicine. Nat. Rev. Mater..

[29] Rezakhani S., Gjorevski N., Lutolf M.P. (2021). Extracellular matrix requirements for gastrointestinal organoid cultures. Biomaterials.

[30] Zhu L., Yuhan J., Yu H. (2023). Decellularized extracellular matrix for remodeling bioengineering organoid's microenvironment. Small.

[31] Guo X., Liu B., Zhang Y. (2024). Decellularized extracellular matrix for organoid and engineered organ culture. J. Tissue Eng..

[32] Li C., An N., Song Q. (2024). Enhancing organoid culture: harnessing the potential of decellularized extracellular matrix hydrogels for mimicking microenvironments. J. Biomed. Sci..

[33] Moura B.S., Monteiro M.V., Ferreira L.P. (2022). Advancing tissue decellularized hydrogels for engineering human organoids. Adv. Funct. Mater..

[34] Brown M., Li J., Moraes C. (2022). Decellularized extracellular matrix: new promising and challenging biomaterials for regenerative medicine. Biomaterials.

[35] Kort-Mascort J., Flores-Torres S., Peza-Chavez O. (2023). Decellularized ECM hydrogels: prior use considerations, applications, and opportunities in tissue engineering and biofabrication. Biomater. Sci..

[36] Giobbe G.G., Crowley C., Luni C. (2019). Extracellular matrix hydrogel derived from decellularized tissues enables endodermal organoid culture. Nat. Commun..

[37] Cho A.-N., Jin Y., An Y. (2021). Microfluidic device with brain extracellular matrix promotes structural and functional maturation of human brain organoids. Nat. Commun..

[38] Shen C., Zhang Z.J., Li X.X. (2023). Intersection of nanomaterials and organoids technology in biomedicine. Front. Immunol..

[39] Nikonorova V.G., Chrishtop V.V., Mironov V.A. (2023). Advantages and potential benefits of using organoids in nanotoxicology. Cells.

[40] Li M., Gong J., Gao L. (2022). Advanced human developmental toxicity and teratogenicity assessment using human organoid models. Ecotoxicol. Environ. Saf..

[41] Mattei F., George J.T., Jolly M.K. (2023). Editorial: organoids, organs-on-chip, nanoparticles and in silico approaches to dissect the tumor-immune dynamics and to unveil the drug resistance mechanisms to therapy in the tumor microenvironment. Front. Immunol..

[42] Septiadi D., Crippa F., Moore T.L. (2018). Nanoparticle–cell interaction: a cell mechanics perspective. Adv. Mater..

[43] Li J., Mao H., Kawazoe N. (2017). Insight into the interactions between nanoparticles and cells. Biomater. Sci..

[44] Chandrakala V., Aruna V., Angajala G. (2022). Review on metal nanoparticles as nanocarriers: current challenges and perspectives in drug delivery systems. Emergent Materials.

[45] Liu Q., Kim Y.-J., Im G.-B. (2021). Inorganic nanoparticles applied as functional therapeutics. Adv. Funct. Mater..

[46] Fallert L., Urigoitia-Asua A., Cipitria A. (2024). Dynamic 3D in vitro lung models: applications of inorganic nanoparticles for model development and characterization. Nanoscale.

[47] Henrique R.B.L., Lima R.R.M., Monteiro C.A.P. (2022). Advances in the study of spheroids as versatile models to evaluate biological interactions of inorganic nanoparticles. Life Sci..

[48] Zhang R., Kiessling F., Lammers T. (2023). Clinical translation of gold nanoparticles. Drug Delivery and Translational Research.

[49] Cunha L., Coelho S.C., Pereira M.d.C. (2022). Nanocarriers based on gold nanoparticles for epigallocatechin gallate delivery in cancer cells. Pharmaceutics.

[50] Kostka K., Sokolova V., El-Taibany A. (2024). The application of ultrasmall gold nanoparticles (2 nm) functionalized with doxorubicin in three-dimensional normal and glioblastoma organoid models of the blood–brain barrier. Molecules.

[51] Park S.B., Cho H.-J., Moon S.R. (2022). Gold nanoparticle-assisted delivery of brain-derived neurotrophic factor to cerebral organoids. Nano Res..

[52] Patino-Guerrero A., Esmaeili H., Migrino R.Q. (2023). Nanoengineering of gold nanoribbon-embedded isogenic stem cell-derived cardiac organoids. RSC Adv..

[53] Caleffi J.T., Aal M.C.E., Gallindo H.d.O.M. (2021). Magnetic 3D cell culture: state of the art and current advances. Life Sci..

[54] Kim J.A., Choi J.-H., Kim M. (2013). High-throughput generation of spheroids using magnetic nanoparticles for three-dimensional cell culture. Biomaterials.

[55] Marovič N., Ban I., Maver U. (2023). Magnetic nanoparticles in 3D-printed scaffolds for biomedical applications. Nanotechnol. Rev..

[56] Ferreira J.N., Hasan R., Urkasemsin G. (2019). A magnetic three-dimensional levitated primary cell culture system for the development of secretory salivary gland-like organoids. J. Tissue Eng. Regen. Med..

[57] Adine C., Ng K.K., Rungarunlert S. (2018). Engineering innervated secretory epithelial organoids by magnetic three-dimensional bioprinting for stimulating epithelial growth in salivary glands. Biomaterials.

[58] Abdel Fattah A.R., Kolaitis N., Van Daele K. (2023). Targeted mechanical stimulation via magnetic nanoparticles guides in vitro tissue development. Nat. Commun..

[59] Lewis J.K., Bischof J.C., Braslavsky I. (2016). The grand challenges of organ banking: proceedings from the first global summit on complex tissue cryopreservation. Cryobiology.

[60] Manuchehrabadi N., Gao Z., Zhang J. (2017). Improved tissue cryopreservation using inductive heating of magnetic nanoparticles. Sci. Transl. Med..

[61] Etheridge M.L., Xu Y., Rott L. (2014). RF heating of magnetic nanoparticles improves the thawing of cryopreserved biomaterials. Technology.

[62] Lee S.-G., Kim J., Seok J. (2024). Development of heart organoid cryopreservation method through Fe3O4 nanoparticles based nanowarming system. Biotechnol. J..

[63] Naidu S., Pandey J., Mishra L.C. (2023). Silicon nanoparticles: synthesis, uptake and their role in mitigation of biotic stress. Ecotoxicol. Environ. Saf..

[64] O'Farrell N., Andrew H., R B., Horrocks (2006). Silicon nanoparticles: applications in cell biology and medicine. Int. J. Nanomed..

[65] Purwada A., Jaiswal M.K., Ahn H. (2015). Ex vivo engineered immune organoids for controlled germinal center reactions. Biomaterials.

[66] Gatto D., Brink R. (2010). The germinal center reaction. J. Allergy Clin. Immunol..

[67] Tan Y., Coyle R.C., Barrs R.W. (2023). Nanowired human cardiac organoid transplantation enables highly efficient and effective recovery of infarcted hearts. Sci. Adv..

[68] Cui D., Kong N., Ding L. (2021). Ultrathin 2D titanium carbide MXene (Ti3C2T) nanoflakes activate WNT/HIF-1α-Mediated metabolism reprogramming for periodontal regeneration. Adv. Healthcare Mater..

[69] Zhao X., Wang L.-Y., Tang C.-Y. (2020). Smart Ti3C2Tx MXene fabric with fast humidity response and joule heating for healthcare and medical therapy applications. ACS Nano.

[70] Chao M., He L., Gong M. (2021). Breathable Ti3C2Tx MXene/Protein nanocomposites for ultrasensitive medical pressure sensor with degradability in solvents. ACS Nano.

[71] Zhang Z., Gao S., Hu Y.-N. (2022). Ti3C2TxMXene composite 3D hydrogel potentiates mTOR signaling to promote the generation of functional hair cells in cochlea organoids. Adv. Sci..

[72] Zhang Y., Dayton P., Yang X. (2014). Toxicity and efficacy of carbon nanotubes and graphene: the utility of carbon-based nanoparticles in nanomedicine. Drug Metab. Rev..

[73] Khajavinia A., El-Aneed A. (2023). Carbon-based nanoparticles and their surface-modified counterparts as MALDI matrices. Anal. Chem..

[74] Ayanda O.S., Mmuoegbulam A.O., Okezie O. (2024). Recent progress in carbon-based nanomaterials: critical review. J. Nanoparticle Res..

[75] Bao L., Cui X., Wang X. (2021). Carbon nanotubes promote the development of intestinal organoids through regulating extracellular matrix viscoelasticity and intracellular energy metabolism. ACS Nano.

[76] Kim N.H., Chae S., Yi S.A. (2023). Peptide-assembled single-chain atomic crystal enhances pluripotent stem cell differentiation to neurons. Nano Lett..

[77] Itoo A.M., Vemula S.L., Gupta M.T. (2022). Multifunctional graphene oxide nanoparticles for drug delivery in cancer. J. Contr. Release.

[78] Shahriari S., Murali S., Santosh P. (2021). Graphene and graphene oxide as a support for biomolecules in the development of biosensors. Nanotechnol. Sci. Appl..

[79] Yildiz G., Bolton-Warberg M., Awaja F. (2021). Graphene and graphene oxide for bio-sensing: general properties and the effects of graphene ripples. Acta Biomater..

[80] Park S., Kim Y.J., Sharma H. (2023). Graphene hybrid inner ear organoid with enhanced maturity. Nano Lett..

[81] Beach M.A., Nayanathara U., Gao Y. (2024). Polymeric nanoparticles for drug delivery. Chem. Rev..

[82] Elmowafy M., Shalaby K., Elkomy M.H. (2023). Polymeric nanoparticles for delivery of natural bioactive agents: recent advances and challenges. Polymers.

[83] Castro K.C.d., Martins C.J., N M.G., Campos (2022). Drug-loaded polymeric nanoparticles: a review. International Journal of Polymeric Materials and Polymeric Biomaterials.

[84] Li X., Sheng S., Li G. (2024). Research progress in hydrogels for cartilage organoids. Adv. Healthcare Mater..

[85] Wang F., Gu Z., Yin Z. (2023). Cell unit-inspired natural nano-based biomaterials as versatile building blocks for bone/cartilage regeneration. J. Nanobiotechnol..

[86] Klemm D., Kramer F., Moritz S. (2011). Nanocelluloses: a new family of nature-based materials. Angew. Chem. Int. Ed..

[87] Thomas B., Raj M.C., K. B A. (2018). Nanocellulose, a versatile green platform: from biosources to materials and their applications. Chem. Rev..

[88] Jonoobi M., Oladi R., Davoudpour Y. (2015). Different preparation methods and properties of nanostructured cellulose from various natural resources and residues: a review. Cellulose.

[89] Nikolits I., Radwan S., Liebner F. (2023). Hydrogels from TEMPO-Oxidized nanofibrillated cellulose support in vitro cultivation of encapsulated human mesenchymal stem cells. ACS Appl. Bio Mater..

[90] Sharma P., Pal V.K., Kaur H. (2022). Exploring the TEMPO-oxidized nanofibrillar cellulose and short ionic-complementary peptide composite hydrogel as biofunctional cellular scaffolds. Biomacromolecules.

[91] Lan X., Ma Z., Szojka A.R.A. (2021). TEMPO-Oxidized cellulose nanofiber-alginate Hydrogel as a bioink for Human Meniscus tissue engineering. Front. Bioeng. Biotechnol..

[92] Isogai A., Saito T., Fukuzumi H. (2011). TEMPO-oxidized cellulose nanofibers. Nanoscale.

[93] Krüger M., Oosterhoff L.A., van Wolferen M.E. (2020). Cellulose nanofibril Hydrogel Promotes Hepatic Differentiation of human liver organoids. Adv. Healthcare Mater..

[94] Curvello R., Garnier G. (2021). Cationic cross-linked nanocellulose-based matrices for the growth and recovery of intestinal organoids. Biomacromolecules.

[95] Curvello R., Kerr G., Micati D.J. (2021). Engineered plant-based Nanocellulose hydrogel for small intestinal organoid growth. Adv. Sci..

[96] Curvello R., Alves D., Abud H.E. (2021). A thermo-responsive collagen-nanocellulose hydrogel for the growth of intestinal organoids. Mater. Sci. Eng. C.

[97] Curvello R., Raghuwanshi V.S., Wu C.M. (2024). Nano- and microstructures of collagen-nanocellulose hydrogels as engineered extracellular matrices. ACS Appl. Mater. Interfaces.

[98] Casalini T., Rossi F., Castrovinci A. (2019). A perspective on polylactic acid-based polymers use for nanoparticles synthesis and applications. Front. Bioeng. Biotechnol..

[99] PLGA-Based Nanoparticles as Cancer Drug Delivery Systems (2014). Asian Pac. J. Cancer Prev. APJCP.

[100] da Luz C.M., Boyles M.S.P., Falagan-Lotsch P. (2017). Poly-lactic acid nanoparticles (PLA-NP) promote physiological modifications in lung epithelial cells and are internalized by clathrin-coated pits and lipid rafts. J. Nanobiotechnol..

[101] Tong Y., Ueyama-Toba Y., Yokota J. (2024). Efficient hepatocyte differentiation of primary human hepatocyte-derived organoids using three dimensional nanofibers (HYDROX) and their possible application in hepatotoxicity research. Sci. Rep..

[102] Xu Y., Fourniols T., Labrak Y. (2022). Surface modification of lipid-based nanoparticles. ACS Nano.

[103] Mehta M., Bui T.A., Yang X. (2023). Lipid-Based nanoparticles for Drug/gene delivery: an overview of the production techniques and difficulties encountered in their industrial development. ACS Mater. Au.

[104] Patel P., Garala K., Singh S. (2024). Lipid-based nanoparticles in delivering bioactive compounds for improving therapeutic efficacy. Pharmaceuticals.

[105] Krause M., Rak-Raszewska A., Naillat F. (2018). Exosomes as secondary inductive signals involved in kidney organogenesis. J. Extracell. Vesicles.

[106] Zhang Y.S., Zhang Y.-N., Zhang W. (2017). Cancer-on-a-chip systems at the frontier of nanomedicine. Drug Discov. Today.

[107] Li D., Zhang R., Le Y. (2024). Organoid-Based assessment of metal–organic framework (MOF) nanomedicines for Ex vivo cancer therapy. ACS Appl. Mater. Interfaces.

[108] Zhang H., Lu Y., Zhang R. (2024). Synthesis of multifunctional plasmonic nanodarts through one-end deposition on gold nanobipyramids for tumor organoid ablation and antimicrobial applications. Adv. Funct. Mater..

[109] McCarthy B., Cudykier A., Singh R. (2021). Semiconducting polymer nanoparticles for photothermal ablation of colorectal cancer organoids. Sci. Rep..

[110] Wang Y., Zhang J., Liu Y. (2024). Realveolarization with inhalable mucus-penetrating lipid nanoparticles for the treatment of pulmonary fibrosis in mice. Sci. Adv..

[111] Poh B.M., Liew L.C., Soh Y.N.A. (2024). MSC-Derived small extracellular vesicles exert cardioprotective effect through reducing VLCFAs and apoptosis in human Cardiac organoid IRI model. Stem Cell..

[112] Kim N.G., Jung D.J., Jung Y.-K. (2023). The effect of a novel mica nanoparticle, STB-MP, on an alzheimer's disease patient-induced PSC-Derived cortical brain organoid model. Nanomaterials.

[113] Astashkina A.I., Jones C.F., Thiagarajan G. (2014). Nanoparticle toxicity assessment using an in vitro 3-D kidney organoid culture model. Biomaterials.

[114] Gerbolés A.G., Galetti M., Rossi S. (2023). Three-Dimensional bioprinting of organoid-based scaffolds (OBST) for long-term nanoparticle toxicology investigation. Int. J. Mol. Sci..

[115] Yang H., Niu S., Guo M. (2024). Applications of 3D organoids in toxicological studies: a comprehensive analysis based on bibliometrics and advances in toxicological mechanisms. Arch. Toxicol..

[116] Issa R., Lozano N., Kostarelos K. (2024). Functioning human lung organoids model pulmonary tissue response from carbon nanomaterial exposures. Nano Today.

[117] Huang Y., Guo L., Cao C. (2022). Silver nanoparticles exposure induces developmental neurotoxicity in hiPSC-derived cerebral organoids. Sci. Total Environ..

[118] Liu L., Wang J., Zhang J. (2023). The cytotoxicity of zinc oxide nanoparticles to 3D brain organoids results from excessive intracellular zinc ions and defective autophagy. Cell Biol. Toxicol..

[119] Mekky G., Seeds M., Diab A.E.-A.A. (2021). The potential toxic effects of magnesium oxide nanoparticles and valproate on liver tissue. J. Biochem. Mol. Toxicol..

[120] Baek A., Kwon I.H., Lee D.-H. (2024). Novel organoid culture System for improved safety assessment of nanomaterials. Nano Lett..

[121] Iqbal M.Z., Luo D., Akakuru O.U. (2021). Facile synthesis of biocompatible magnetic titania nanorods for T1-magnetic resonance imaging and enhanced phototherapy of cancers. J. Mater. Chem. B.

[122] Leite P.E.C., Pereira M.R., Harris G. (2019). Suitability of 3D human brain spheroid models to distinguish toxic effects of gold and poly-lactic acid nanoparticles to assess biocompatibility for brain drug delivery. Part. Fibre Toxicol..

[123] Bloem B.R., Okun M.S., Klein C. (2021). Parkinson's disease. Lancet.

[124] Tolosa E., Garrido A., Scholz S.W. (2021). Challenges in the diagnosis of Parkinson's disease. Lancet Neurol..

[125] Calabresi P., Di Lazzaro G., Marino G. (2023). Advances in understanding the function of alpha-synuclein: implications for Parkinson's disease. Brain.

[126] Lee M.-H., Liu K.-T., Thomas J.L. (2020). Peptide-Imprinted Poly(hydroxymethyl 3,4-ethylenedioxythiophene) nanotubes for detection of α Synuclein in human brain organoids. ACS Appl. Nano Mater..

[127] Cui K., Chen W., Cao R. (2022). Brain organoid-on-chip system to study the effects of breast cancer derived exosomes on the neurodevelopment of brain. Cell Regen..

[128] Eivazzadeh-Keihan R., Sadat Z., Lalebeigi F. (2024). Effects of mechanical properties of carbon-based nanocomposites on scaffolds for tissue engineering applications: a comprehensive review. Nanoscale Adv..

[129] Saheli M., Sepantafar M., Pournasr B. (2018). Three-dimensional liver-derived extracellular matrix hydrogel promotes liver organoids function. J. Cell. Biochem..

[130] Willemse J., van Tienderen G., van Hengel E. (2022). Hydrogels derived from decellularized liver tissue support the growth and differentiation of cholangiocyte organoids. Biomaterials.

[131] Chen J., Ma S., Yang H. (2023). Generation and metabolomic characterization of functional ductal organoids with biliary tree networks in decellularized liver scaffolds. Bioact. Mater..

[132] Kim S., Min S., Choi Y.S. (2022). Tissue extracellular matrix hydrogels as alternatives to Matrigel for culturing gastrointestinal organoids. Nat. Commun..

[133] Garreta E., Moya-Rull D., Marco A. (2024). Natural hydrogels support kidney Organoid generation and promote in vitro angiogenesis. Adv. Mater..

[134] Kim J.W., Nam S.A., Yi J. (2022). Kidney decellularized extracellular matrix enhanced the vascularization and maturation of human kidney organoids. Adv. Sci..

[135] Nag S., Boyd A.S. (2023). Decellularization of mouse kidneys to generate an extracellular matrix gel for Human induced pluripotent stem cell derived renal organoids. Organ.

[136] Simsa R., Rothenbücher T., Gürbüz H. (2021). Brain organoid formation on decellularized porcine brain ECM hydrogels. PLoS One.

[137] Wu W., Liu Y., Liu R. (2024). Decellularized brain extracellular matrix hydrogel aids the Formation of human spinal-cord organoids recapitulating the complex three-dimensional Organization. ACS Biomater. Sci. Eng..

[138] Wang Z., Liu R., Liu Y. (2024). Human Placenta decellularized extracellular Matrix Hydrogel promotes the generation of human spinal cord organoids with dorsoventral Organization from human induced pluripotent stem cells. ACS Biomater. Sci. Eng..

[139] Milton L.A., Davern J.W., Hipwood L. (2024). Liver click dECM hydrogels for engineering hepatic microenvironments. Acta Biomater..

[140] Chan W.W., Yu F., Le Q.B. (2021). Towards biomanufacturing of cell-derived matrices. Int. J. Mol. Sci..

[141] Hoshiba T. (2021). Cultured cell-derived decellularized extracellular matrix (cultured cell-derived dECM): future applications and problems — a mini review. Current Opinion in Biomedical Engineering.

[142] Jin H., Xue Z., Liu J. (2024). Advancing organoid engineering for tissue regeneration and biofunctional reconstruction. Biomater. Res..

[143] Shao Y., Wang J., Jin A. (2025). Biomaterial-assisted organoid technology for disease modeling and drug screening. Mater. Today Bio.

[144] McWilliam R.H., Chang W., Liu Z. (2023). Three-dimensional biofabrication of nanosecond laser micromachined nanofibre meshes for tissue engineered scaffolds. Biomater. Transl..

[145] Song S., Zhang J., Fang Y. (2025). Nerve–bone crosstalk manipulates bone organoid development and bone regeneration: a review and perspectives. Organoid Research.

[146] Zhao J., Shen F., Sheng S. (2025). Cartilage-on-chip for osteoarthritis drug screening. Organoid Research.

[147] Xu Z.-Y., Wang M., Shi J.-Y. (2025). Engineering a dynamic extracellular matrix using thrombospondin-1 to propel hepatocyte organoids reprogramming and improve mouse liver regeneration post-transplantation. Mater. Today Bio.

[148] Xu Z.Y., Huang J.J., Liu Y. (2023). Extracellular matrix bioink boosts stemness and facilitates transplantation of intestinal organoids as a biosafe Matrigel alternative. Bioeng. Transl. Med..

[149] Choi Y.M., Lee H., Ann M. (2023). 3D bioprinted vascularized lung cancer organoid models with underlying disease capable of more precise drug evaluation. Biofabrication.

[150] Kim J., Kim J., Gao G. (2024). Bioprinted organoids platform with tumor vasculature for implementing precision personalized medicine targeted towards gastric cancer. Adv. Funct. Mater..

[151] Liu H., Gan Z., Qin X. (2024). Advances in microfluidic technologies in organoid research. Adv. Healthcare Mater..

[152] Saorin G., Caligiuri I., Rizzolio F. (2023). Microfluidic organoids-on-a-chip: the future of human models. Semin. Cell Dev. Biol..

[153] Park B., Park J., Han S. (2025). Advances in organoid-on-a-chip for recapitulation of human physiological events. Mater. Today.

[154] Chauhdari T., Zaidi S.A., Su J. (2025). Organoids meet microfluidics: recent advancements, challenges, and future of organoids-on-chip.

[155] Jin Y., Kim J., Lee J.S. (2018). Vascularized liver organoids generated using induced hepatic tissue and dynamic liver-specific microenvironment as a drug testing platform. Adv. Funct. Mater..

[156] Zhu L., Yuhan J., Yu H. (2023). Decellularized extracellular matrix for remodeling bioengineering organoid's microenvironment. Small.

[157] Kuşoğlu A., Yangın K., Özkan S.N. (2023). Different decellularization methods in bovine lung tissue reveals distinct biochemical composition, stiffness, and viscoelasticity in reconstituted hydrogels. ACS Appl. Bio Mater..

[158] Spagnol G., Sensi F., De Tommasi O. (2023). Patient derived organoids (PDOs), extracellular matrix (ECM), tumor microenvironment (TME) and drug screening: state of the art and clinical implications of ovarian cancer organoids in the era of precision medicine. Cancers.

[159] van Tienderen G.S., Rosmark O., Lieshout R. (2023). Extracellular matrix drives tumor organoids toward desmoplastic matrix deposition and mesenchymal transition. Acta Biomater..

[160] Ferreira L.P., Gaspar V.M., Mendes L. (2021). Organotypic 3D decellularized matrix tumor spheroids for high-throughput drug screening. Biomaterials.

[161] Jin H., Yang Q., Yang J. (2024). Exploring tumor organoids for cancer treatment. APL Mater..

[162] van Tienderen G.S., van Beek M.E.A., Schurink I.J. (2023). Modelling metastatic colonization of cholangiocarcinoma organoids in decellularized lung and lymph nodes. Front. Oncol..

[163] Chen Z., Long L., Wang J. (2024). Constructing Tumor organoid-like tissue for reliable drug screening using liver-decellularized extracellular Matrix scaffolds. ACS Omega.

[164] Varinelli L., Guaglio M., Brich S. (2022). Decellularized extracellular matrix as scaffold for cancer organoid cultures of colorectal peritoneal metastases. J. Mol. Cell Biol..

[165] Tevlek A. (2024). The role of decellularized cell derived extracellular matrix in the establishment and culture of in vitro breast cancer tumor model. Biomed. Mater..

[166] Kim M., Kang D., Han H. (2025). Light-activated decellularized extracellular matrix-based bioinks for enhanced mechanical integrity. Mater. Today Bio.

[167] Ostadi Y., Khanali J., Tehrani F.A. (2024). Decellularized extracellular matrix scaffolds for soft tissue augmentation: from host–scaffold interactions to bottlenecks in clinical translation. Biomater. Res..

[168] Biehl A., Gracioso Martins A.M., Davis Z.G. (2023). Towards a standardized multi-tissue decellularization protocol for the derivation of extracellular matrix materials. Biomater. Sci..

[169] Gai T., Zhang H., Hu Y. (2025). Sequential construction of vascularized and mineralized bone organoids using engineered ECM-DNA-CPO-based bionic matrix for efficient bone regeneration. Bioact. Mater..

[170] Qiu H., Zhang L., Wang D. (2022). Silver nanoparticles improve the biocompatibility and reduce the immunogenicity of xenogeneic scaffolds derived from decellularized pancreas. Cell. Reprogr..

[171] Yuan Y., Xu Y., Mao Y. (2024). Three birds, one stone: an osteo-microenvironment stage-regulative scaffold for bone defect repair through modulating early Osteo-Immunomodulation, middle neovascularization, and later osteogenesis. Adv. Sci..

[172] Kim J., Jang J., Cho D.-W. (2021). Controlling cancer cell behavior by improving the stiffness of gastric tissue-decellularized ECM bioink with cellulose nanoparticles. Front. Bioeng. Biotechnol..

[173] Abdel Fattah A.R., Kolaitis N., Van Daele K. (2022). Local actuation of organoids by magnetic nanoparticles. bioRxiv.

[174] Ly P.-D., Ly K.-N., Phan H.-L. (2024). Recent advances in surface decoration of nanoparticles in drug delivery. Front. Nanotechnol..

[175] Costoya J., Surnar B., Kalathil A.A. (2022). Controlled release nanoplatforms for three commonly used chemotherapeutics. Mol. Aspect. Med..

[176] Subbotina J., Rouse I., Lobaskin V. (2023). In silico prediction of protein binding affinities onto core–shell PEGylated noble metal nanoparticles for rational design of drug nanocarriers. Nanoscale.

[177] Bai L., Wu Y., Li G. (2024). AI-enabled organoids: construction, analysis, and application. Bioact. Mater..

[178] Smirnova L., Caffo B.S., Gracias D.H. (2023). Organoid intelligence (OI): the new frontier in biocomputing and intelligence-in-a-dish. Front. Sci..

